# Serum immunoglobulin and the threshold of Fc receptor-mediated immune activation

**DOI:** 10.1016/j.bbagen.2023.130448

**Published:** 2023-08-29

**Authors:** Hannah Bauer-Smith, Abigail S.L. Sudol, Stephen A. Beers, Max Crispin

**Affiliations:** a School of Biological Sciences, University of Southampton, Southampton SO17 1BJ, UK; b Centre for Cancer Immunology, School of Cancer Sciences, University of Southampton Faculty of Medicine, Southampton SO16 6YD, UK

**Keywords:** Antibody structure, Glycosylation, Effector functions, Immunoglobulin, Therapeutic antibodies, Fc, Fc receptors

## Abstract

Antibodies can mediate immune recruitment or clearance of immune complexes through the interaction of their Fc domain with cellular Fc receptors. Clustering of antibodies is a key step in generating sufficient avidity for efficacious receptor recognition. However, Fc receptors may be saturated with prevailing, endogenous serum immunoglobulin and this raises the threshold by which cellular receptors can be productively engaged. Here, we review the factors controlling serum IgG levels in both healthy and disease states, and discuss how the presence of endogenous IgG is encoded into the functional activation thresholds for low- and high-affinity Fc receptors. We discuss the circumstances where antibody engineering can help overcome these physiological limitations of therapeutic antibodies. Finally, we discuss how the pharmacological control of Fc receptor saturation by endogenous IgG is emerging as a feasible mechanism for the enhancement of antibody therapeutics.

## 1. Introduction

Antibody engagement with Fc receptors (FcRs) expressed on myeloid cells is important for mediating pathogen clearance and in the mechanism of action of therapeutic antibodies, such as those against cancerous cells. However, the function of these antibodies can be impeded by the presence of endogenous antibodies which act as competitors for the antibody receptors on immune effector cells. The evidence that this competition can have a significant impact on the function of antibodies has been shown in cellular assays, whereby competing antibody both controls the amount of on target antibody required for activity and can completely limit immune cell function when target density is low [1]. Moreover, the presence of endogenous antibody has also been demonstrated to potently limit therapeutic antibody function in animal models of breast cancer, colon cancer and lymphoma [2]. Finally in humans, endogenous antibody concentration has been shown to have a significant impact on immune complex (IC) clearance rates *in vivo* [3], and *ex vivo* assessment of phagocytosis [2]. In this review, we explore the parameters influencing the threshold of immune activation, with the emphasis on these competition effects.

**Fig. 1.**
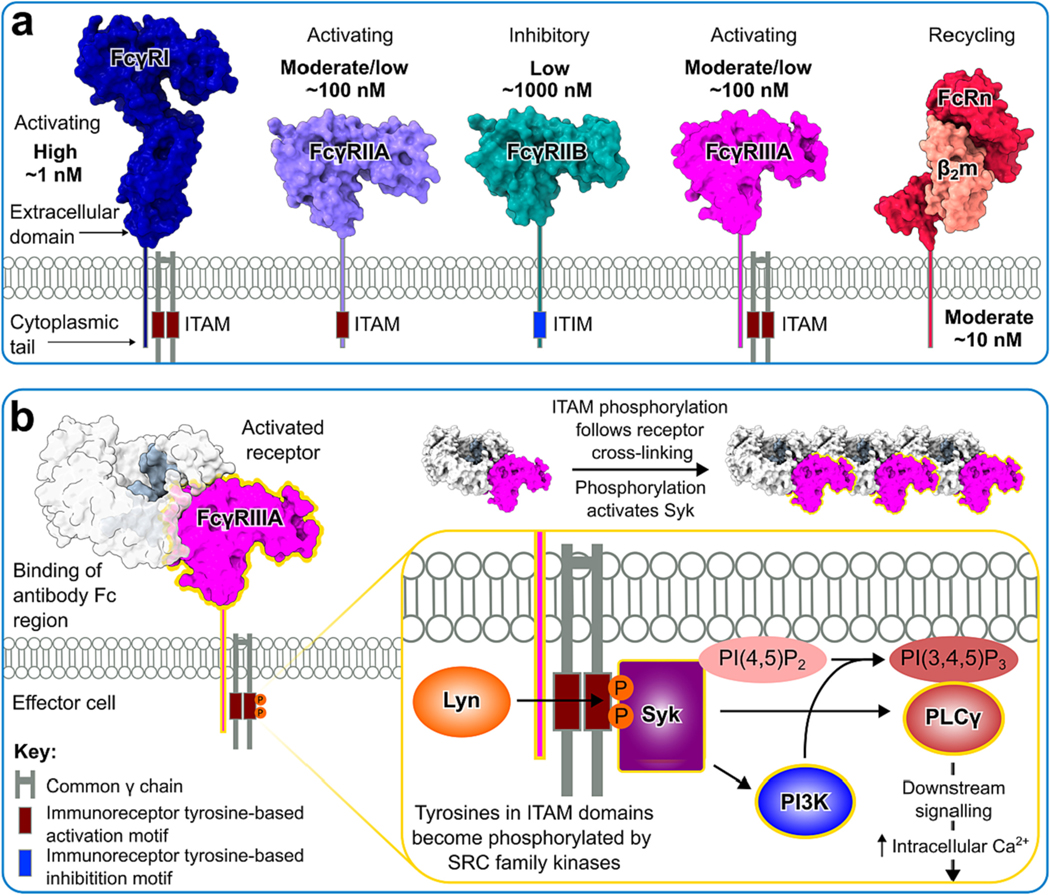
Structure and function of human Fc gamma receptors. a Structures of FcγRs and the neonatal Fc receptor (PDB codes 4W4O, 1H9V, 3WJJ, 5XJE and 7Q15 for FcγRs I, IIA, IIB, IIIA and FcRn, respectively). FcγRI and FcγRIIIA require association with the common γ-chain to initiate signalling. Receptors display variation in their affinity to IgG Fc, as indicated. b Signalling *via* ITAM domains. Tyrosine residues in ITAM motifs become phosphorylated by SRC family kinases, such as Lyn, following cross-linking of cell surface FcγRs by IgG-immune complexes, subsequently leading to Syk activation. This results in downstream activation of phospholipase C gamma 1 (PLCγ), which activates further downstream signalling, leading to increased levels of intracellular calcium and, ultimately, immune cell activation. Stimulation of phosphoinositide 3 kinase (PI3K) catalyses phosphorylation of PI(4,5)P_2_ into PI(3,4,5)P_3_ present in the plasma membrane, which serves as a docking site for PLCγ, thus recruiting it to the membrane and promoting further phosphorylation and activation.

Activation of immune cells is a tightly regulated process that depends on the integration of various extracellular and intracellular signalling pathways to form an appropriate response. Activating FcRs signal *via* intracellular domains containing immunoreceptor tyrosine-based activation motifs (ITAM) either directly, or in association with accessory proteins (Fig. 1). A characteristic of the activation of these receptors is the requirement for multiple ligand-receptor interactions to enable crosslinking and receptor aggregation at the surface of the cell, which subsequently initiates phosphorylation of the ITAMs and downstream signalling [4–6]. The presence of inhibitory FcRs featuring immunoreceptor tyrosine-based inhibition motifs (ITIMs) which can impede ITAM signalling combined with the requirement for receptor aggregation creates a threshold that prevents inappropriate immune cell activation and inflammation [7,8].

Antibodies, or immunoglobulins, are glycoproteins which stimulate the ITAM signalling pathway upon crosslinking with activating FcRs expressed by immune effectors. They are comprised of two light chains and two heavy chains which assemble to form a ‘Y’ shaped structure that is divided into two domains connected by a flexible hinge; the bivalent antigen binding F(ab’)_2_ domain, and the Fc domain that interacts with FcRs. Antibody Fc domains are glycosylated which can help protein folding and assembly. Moreover, Fc glycans may also influence Fc receptor interactions [9,10] and antibody clearance rates [11–14].

Two classes of light chain exist, namely κ and λ, which associate with the heavy chain during antibody assembly. There are five classes of heavy chain in humans due to variation in the polypeptide sequence; namely α, δ, ε, γ, and μ, which in turn give rise to the immunoglobulin isotypes IgA, IgD, IgE, IgG and IgM respectively. There are four IgG subclasses (IgG1, IgG2, IgG3 and IgG4) and two classes of IgA (IgA1 and IgA2) which exhibit different effector functions. Notable structural differences between the isotypes occur in the hinge region and the carboxy terminus. In IgA, IgD and IgG the Fabs and Fc domain are connected by a peptidic hinge region that in the case of IgA1 and IgD can be modified by O-linked glycosylation [15,16]. In contrast, the hinge in IgE and IgM is replaced by an immunoglobulin constant domain which is capable of mediating extensive conformational flexibility [17,18]. Furthermore, IgA and IgM feature a tailpiece sequence which enables the formation of antibody dimers or pentamers respectively, through the interaction with an accompanying J-chain [19]. Fundamentally, this structural variation across the isotypes is related to the different functions (discussed in the following section) and is reflective of their anatomical location, abundance in the sera and the stage at which they appear during an immune response. The functional capacity exhibited by antibodies means that they are frequently used as immunotherapeutic interventions for the treatment of diseases, ranging from cancers and infections, to autoimmunity, allergies and migraines. Antibody-based therapeutics include intravenous immunoglobulin (IVIg), monoclonal antibodies (mAbs), and antibody-like proteins (ALPs, Table 1) which may be designed with or without a functional Fc domain depending on their proposed mechanism of action.

In circumstances when Fc-FcR engagement is necessary for the mechanism of action of the administered therapy, competing non-specific antibodies may hinder Fc-FcR interactions and impede FcR signalling. Understanding the importance of antibody competition requires an appreciation of how antibodies, both endogenous and therapeutic, interact with the immune system under a range of normal physiological conditions and how dysregulation of this complex regulatory network may contribute to disease pathogenesis. The importance of Fc-FcR interactions is well illustrated by the complex mechanism of action of IVIg and mAbs, discussed in section 4: The signal-to-noise problem in IgG signalling. In the present review, we will discuss how variation in epitope selection and density, Fc glycan composition, inheritance of specific FcR polymorphisms, and IgG subclass distribution can be impacted by competing endogenous antibody in states of disease. Finally, we shall explore how various antibody engineering techniques may be adopted to overcome these physiological limitations.

## 2. Immune recognition of antibodies

One function of antibodies is the recognition and clustering of target antigen into an immune complex (IC). Within these ICs, the antibody Fc domain mediates various effector functions by engagement with complement proteins in the sera and FcRs present on immune cells populations such as monocytes, macrophages and natural killer (NK) cells. The affinity between antibody Fc and FcR is measured by the equilibrium dissociation constant ($K_{\text{D}}$), which is a ratio of the rate of dissociation ($k_{\text{d}}$) against rate of association ($k_{\text{a}}$). Since the value of $K_{\text{D}}$ varies between individual FcRs, some may be less accessible to mAbs or ALPs than others due to saturation by circulating endogenous antibody, and therefore more difficult to stimulate a functional response on the immune cell on which they are expressed. Despite this saturation of low affinity receptors by endogenous antibody, they can be rapidly displaced by IC interactions, whereas high affinity receptors act to limit antibody exchange. These competition effects do however act to modulate the threshold of immune activation.

An understanding of immune thresholds may be informed by our knowledge of immune receptors (Fig. 1). Canonical Fc receptors include the Fcγ receptors (FcγRI, FcγRIIA, FcγRIIB, FcγRIIIA, FcγRIIIB,), FcεRI, FcαRI, Fcα/μR and FcμR while the while noncanonical Fc receptors include the neonatal FcR (FcRn) and FcεRII [33]. The role of FcRn in IgG biodistribution and catabolism will be discussed in the following section. All mentioned canonical Fc receptors, with the exception of FcγRIIB and FcγRIIIB, are considered activating and are therefore capable of triggering an immune response in the cell on which they are expressed. Stimulation of the activating receptors may lead to association with the FcR common γ-chain (FcRγ), which possess a conserved ITAM in their cytoplasmic tails [34,35]. In contrast, FcγRIIA signals independently of FcRγ as it possess an ITAM in its cytoplasmic domain [36] (Fig. 1a). Notably, FcγRI is the only FcγR that is capably of forming stable, long-lived interactions with IgG, owing to its high affinity for Fc.

Because interactions between the low affinity FcγRs and monomeric IgG is weak (1000–100 nM), successful cross-linking of the receptors and subsequent ITAM stimulation depends on Fc avidity that arises following opsonisation of a pathogen. The avidity effect is fundamental to many biological processes which depend on multiple weak interactions and describes how stable complexes can be formed by proximity effects driving binding despite the individual interacting partners exhibiting low affinity [37]. Notably, this allows multiple Fc-FcγR interactions to occur and facilitates FcγR clustering at the surface of the immune cell, enabling ITAM stimulation and initiation of downstream signalling pathways (Fig. 1b). This avidity effect combined with the necessity of FcR clustering needed to trigger an immune response [4,38,39] ultimately creates an activation threshold that may further be influenced by the interplay between ITAM and ITIM signalling. The consequence of endogenous serum IgG on Fc-mediated effector functions is illustrated by the concentration-dependent impact of serum IgG on immune complex clearance [3].

In addition to the impact of receptor diversity in controlling immune thresholds, IgG also has multiple isotypes with different effector functions. IgG antibodies predominate in human sera, representing approximately 70% of the total antibody content, and may be further classified into four subclasses: IgG1, IgG2, IgG3 and IgG4, which are named in accordance with their abundance. The main structural differences between the different subclasses occur in the hinge region, which differ in both length (IgG3 > IgG1 > IgG2 = IgG4) and the number of disulphide bonds (IgG3 *>*IgG2 *>*IgG1 = IgG4). IgG1 and IgG3 are more effective at fixing complement [40,41], and exhibit higher affinity for the various FcγRs than IgG2 or IgG4 [42]. Notably, high concentrations of IgG *in vivo* results in competition between individual IgGs for FcγR engagement, the consequences of which will be discussed in the following section.

An understanding of the ITAM signalling pathway is important due to complex interplay between the activating and inhibitory receptors. Cross-linking of cell surface FcγRs by IgG-immune complexes stimulates phosphorylation of tyrosine residues in ITAM by SRC family protein kinases such as the tyrosine kinase Lyn [35,43,44], which subsequently leads to Syk activation. The downstream events of Syk activation includes activation of Phospholipase C gamma 1 (PLCγ) which stimulates a downstream signalling pathway leading to increased levels of intracellular calcium and subsequent cell activation. Stimulation of phosphoinositide 3 kinase (PI3K) catalyses the phosphorylation of PI(4,5)P_2_ into PI(3,4,5)P_3_ in the plasma membrane which serves as a docking site for PLCγ, thus recruiting them at the plasma membrane and promoting further phosphorylation and activation (Fig. 1b). Notably, this activation pathway can be inhibited by co-aggregation of FcγRIIB, which contains an intracellular tyrosine inhibitory motif (ITIM) that impedes activating signalling (Fig. 1a). However, more recent evidence has shown that the inhibitory effect of FcγRIIB is not always dependent on ITIM stimulation [45], which will be discussed further in section 6: The inhibitory nature of FcγRIIB. Other ITIM-containing receptors have also been found to co-aggregate with the activating FcRs, such as Sialic acid-binding Ig-like lectins (Siglecs) and Ig-like transcripts (ILTs) [46]. As the name would suggest, the natural ligand for Siglecs is sialic acid which is ubiquitously expressed on human cells. In particular, Siglec-3 (CD33) has been shown to reduce FcγRI responses in monocyte cell lines, which was found to be dependent on SHP-1 recruitment to the cytoplasmic domain [47].

The functional consequence of ITAM stimulation will vary depending on the isotype of the interacting antibody, the FcR engaged, and the type of immune cell on which it is expressed. IgA is the most prevalent antibody isotype found at mucosal surfaces, where it exists predominantly in the form of secretory IgA comprised of dimeric IgA, J-chain and secretory component. There are two isotypes of IgA, IgA1 and IgA2, both of which serves to neutralise pathogens [48]. IgA1 is the most abundant isotype in the serum, while IgA2 is often more prevalent at mucosal surfaces [48]. Monomeric IgA is reported to circulate at concentrations of 0.7–4 mg/mL (4–25 μM) within the serum [49] and although it is lower at mucosal surfaces, an accurate assessment of concentration is complicated by complex biodistribution systems. Its receptor, FcαRI, is expressed on neutrophils, eosinophils, monocytes, macrophages and Kupffer cells [50–52], and binds monomeric IgA1 with an affinity of 150 nM [53] which may indicate that this receptor is saturated on immune cell populations. Of interest, there is evidence that transient interactions between monomeric IgA and FcαRI lead to inhibitory signalling [54]. IgA in immune complex (IC), however, binds FcαRI with high affinity due to increased avidity between FcαRI and Fc. Engagement between FcαRI and IC may cause cross-linking of the receptor which can trigger antibody dependent cellular phagocytosis (ADCP) [55,56], or the release of neutrophil extracellular traps (NETs) in response to cell death (often referred to NETosis) [56,57] of the opsonised target. Cross-linking of FcαRI in neutrophil populations may also trigger antibody-dependent cellular trogocytosis (ADCT) [58], which describes an immune effector ‘gnawing’ the plasma membrane of a target cell [59,60].

Monomeric IgE is notable for exhibiting low abundance in human serum and displaying high affinity to FcεRI ($K_{\text{D}}$= 1 nM) [61]. Despite circulating at a low concentration (5 and 3000 ng/mL; 33–20,000 nM), the affinity is sufficiently high that the receptor remains saturated [62,63], thus enabling IgE effector cells such as basophils and mast cells to remain stably loaded with IgE specificities. Receptor-bound IgE requires interaction with multivalent antigen to enable FcεRI crosslinking. Subsequent downstream signalling induces the secretion of inflammatory mediators, such as histamine, in basophils and mast cell populations. Of note, FcεRI is also expressed by monocytes and dendritic cells (DCs) although its function in these contexts is less well understood.

Overall, the immune recognition of antibodies involves a range of cellular receptors which vary in their affinity to antibodies. The prevailing concentration of antibodies together with Fc receptor affinity dictates the degree of receptor saturation, therefore local variation in antibody concentration influences Fc receptor accessibility and the threshold of immune activation. Moreover, stimulation of ITIM signalling, or lack thereof, may further regulate the induction of immune responses.

## 3. Synthesis, catabolism and biodistribution of antibodies

Understanding the metabolism of antibodies is an important parameter in assessing the impact of local antibody concentration on immunological signalling thresholds. Antibodies are produced by plasma cells in response to antigen stimulation, which is thought to be the principle driver of antibody synthesis [64]. When the B-cell receptor (BCR) encounters antigen within the germinal centre they will proliferate, undergo affinity maturation and hypermutate the variable regions of their immunoglobulin encoding genes, leading to the production of high affinity antibodies. B cells can be stimulated in a T-cell independent manner whereby multivalent antigens can drive BCR clustering. Alternatively, B cells can be stimulated by helper T cells that have been themselves stimulated through professional antigen presenting cells (APCs) equipped with innate immune receptors. The antibody-secreting plasma cells may become long-lived plasma cells (LLPCs) which persist for decades once the initial pathogen is cleared [65,66]. Factors driving LLPC longevity and survival are not well understood, but have been extensively reviewed by Lightman et al. [67]. The LLPCs are thought to reside in the bone marrow and maintain antibody production [68,69] even without the need for antigen re-exposure [70]. They mainly produce IgG and IgA immunoglobulins [71], which may provide rationale as to why these isotypes are the most abundant in the serum. Additionally, the rate at which IgG specifically undergoes catabolism is influenced by its concentration in the serum, with evidence that the half-life of IgG is prolonged in those with low levels of circulating IgG [72–74]. However, the same phenomenon has not been observed for IgA or IgM isotypes [75].

**Fig. 2.**
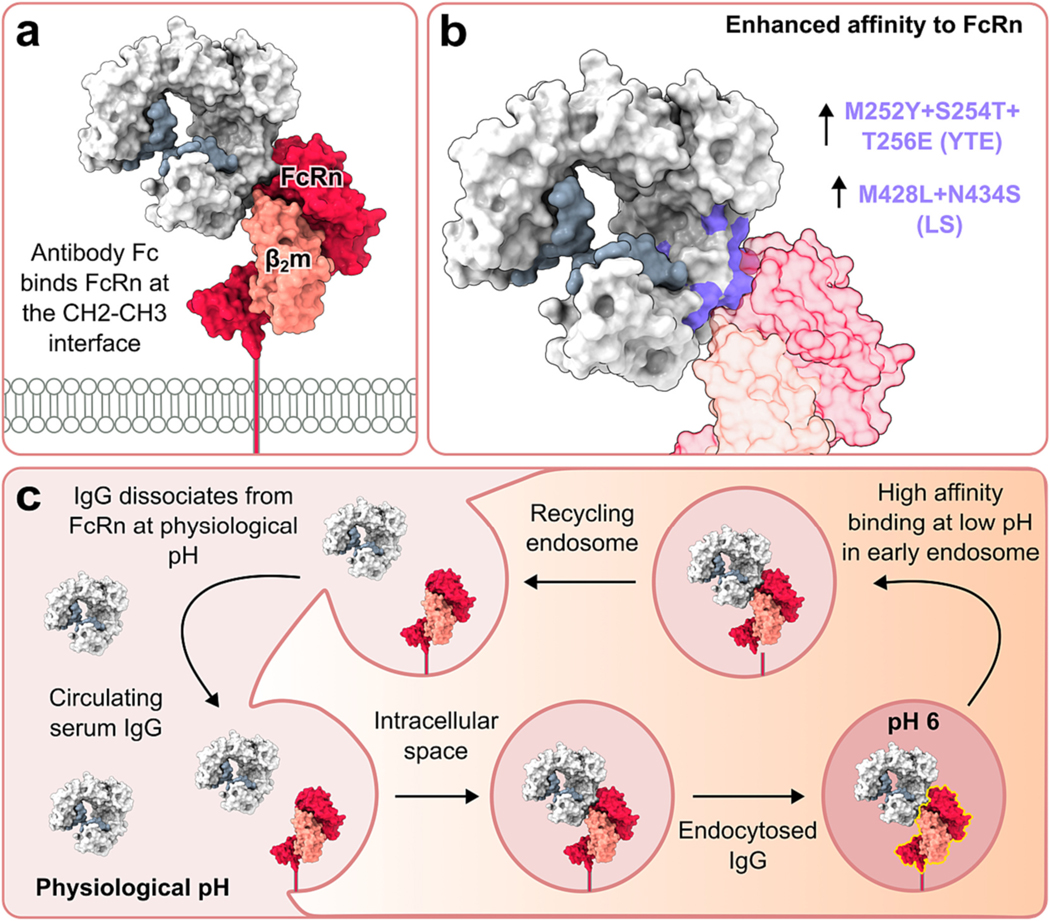
FcRn and biodistribution of IgG. a Crystal structure of IgG Fc bound to human FcRn (PDB code 7Q15) shows how the receptor binds IgG Fc at the interface between its Cγ2 and Cγ3 domains. b Mutations in IgG Fc which affect binding affinity to FcRn, which are currently being utilised in the clinic [87,88]. IgG Fc residues implicated in FcRn binding are coloured purple. c FcRn regulates the *in vivo* persistence of IgG and enables biodistribution of IgG within tissues. IgG can be salvaged in early or sorting endosomes from lysosomal degradation, due to increased binding affinity for FcRn at acidic, endosomal pH (pH 6). (For interpretation of the references to colour in this figure legend, the reader is referred to the web version of this article.)

Susceptibility of IgG catabolism to changing concentration levels, coined the concentration-catabolism effect, is largely attributable to interactions with the neonatal Fc receptor (FcRn, Fig. 2) which recycles pinocytosed IgG back into the circulation. As summarised by Ghetie and Ward, ‘As the serum IgG levels rise, the protective receptors become saturated and more IgG is destined for degradation following uptake’ [76]. The transfer of IgG from mother to foetus is also mediated by FcRn which provides infants with passive humoral immunity [77–80]. The presence of FcRn ultimately enables IgG salvage from the lysosome following pinocytic uptake, thus increasing its half-life. The relatively high affinity that exists between IgG Fc and FcRn at pH 6 enables binding to occur in the early or sorting endosomes. IgG can then be trafficked back to the cell surface in recycling compartments and released from FcRn during exocytosis into the extracellular space following exposure to neutral pH [81–86] (Fig. 2c).

The systemic pharmacokinetics of IgG may be influenced by FcRn due to its role in mediating IgG recycling, degradation and distribution within a given tissue. Indeed, it is well established that IgG half-life *in vivo* can be influenced by mutations that impact Fc binding affinity for FcRn [87–95] (Fig. 2b). The importance of FcRn on IgG homeostasis is also supported by the finding that IgG undergoes hyper-catabolism and decreased serum concentration levels in FcRn-deficient mice [94,96,97]. There is also evidence that antibody clearance levels can be restored to normal levels by IgG reconstitution in mice with low FcRn expression [98]. This effect was attributed to a reduction IgG internalisation mediated by mFcγRII and will be discussed in more detail in section 6: The inhibitory nature of FcγRIIB.

Our knowledge of FcRn and the role it plays in IgG serum concentrations can further our understanding of the complexities of antibody biodistribution and catabolism in local environments. The expression of FcRn has been characterised in various tissue types, such as the vascular endothelium (including those that comprise the blood brain barrier (BBB) [99]) and tissue resident macrophages (Kupffer cells within the liver, alveolar macrophages within the lungs and intestinal macrophages [100]). Such FcRn-expressing cell types may be responsible for mediating influx and efflux of IgG within the organ in which they reside, with evidence that FcRn-mediated transcytosis can impact IgG deposition into the tissues [101–103]. The role of FcRn in salvaging IgG from lysosomal degradation means that varying FcRn expression levels across different tissues may also impact local catabolism [101,103,104].

A comprehensive, physiologically based pharmacokinetic (PBPK) model has been developed by Garg et al. to characterize IgG deposition in plasma and in tissues [101]. The tissues modelled in this study were subdivided into vascular, endosomal and interstitial spaces, and incorporated parameters such as blood circulation, and IgG uptake, recycling and catabolism based upon data previously described in the literature. Values obtained from this PBPK model were then assessed against observed values from wild-type (WT) and FcRn knock-out (KO) murine models which were injected with radiolabelled mAbs. The resulting model predicted that the skin, muscle, liver, and gut are the major organs responsible for IgG catabolism, accounting for approximately 33, 24, 16, and 12% of the total IgG elimination, which may be reflective of organ size. It was also demonstrated that tissue IgG exposure was reduced in FcRn KO murine models, with significant decreases observed in the skin and muscle, which may indicate that they depend on FcRn for their IgG distribution.

A separate biodistribution study conducted by Chen et al. also reported a reduction in the IgG tissue-to-blood (T/B) exposure ratio associated with the skin, lymph node, muscle and adipose tissue of FcRn KO murine models. This may be indicate that IgG salvage or IgG influx into such tissues is mediated by FcRn [102]. The same study also observed an increase in T/B ratio in the liver, spleen, kidney and lung which may suggest these organs depend on FcRn expression to mediate IgG efflux into the plasma. Notably, IgG catabolism in the liver may also be contributing to the high rates of IgG exposure [103]. Overall, both studies by Garg et al. and Chen et al. support the notion that FcRn is required to enable IgG distribution within the skin and muscle. However, conflicting findings for the role of FcRn in other tissues may be attributable to differences in the species of IgG (human *vs* murine) or the differing FcRn KO models (α chain FcRn KO *vs* the β2-microglobulin FcRn KO) used by Garg et al. and Chen et al. respectively.

The role of FcRn in IgG catabolism in individual tissues has also been explored [103]. Radiolabelled IgG with decreased binding affinity to FcRn was injected into mice and monitored for differences in tissue uptake compared to WT IgG. As expected, plasma concentrations of the FcRn-null antibody declined more rapidly than the WT version. The area under the tissue concentration-time curve between 0 and 7 days suggested that that the spleen and liver were major sites of IgG catabolism in the absence of FcRn protection. These organs also accounted for the largest proportion of IgG catabolism in the presence of FcRn protection, but other organs such as lungs, kidney, heart and skin were also found to be contributing. It should be noted that these figures are reported on a tissue mass-normalised basis, and that the skin may be the most important in terms of absolute mass since it accounts for a larger percentage of body weight. These results differ somewhat compared to Garg et al., whose data suggested a more significant role of muscle contribution to catabolism rather than spleen. It is possible that such discrepancies arose due to the use of different radioactive labels in these studies, with the use of the residualizing indium-111 by Yip et al. compared to Garg et al. who use the non-residualizing iodine-125. Since residualizing labels may be detected following antibody catabolism, this may have inflated the contribution of the spleen as major site of catabolism.

These studies using radiolabelled mAbs [101–103] have, however, received some criticism [105]. This is due to the nature of the labels used, which may influence the physiochemical properties of the mAb [106–108], dissociate from the mAb during the experiment [107] or even cause preferential accumulation in certain tissues depending on the label used [107,109]. These studies also opted to analyse whole tissue concentrations of IgG, and do not distinguish between the various compartments within the tissue, such as cellular, vascular or interstitial space. As such, the assessment of antibody concentrations in different tissue micro-environments is hampered by experimental complexities and therefore this limits our ability to fully assess FcγR receptor saturation effects. Nonetheless, these studies provide evidence for the role of FcRn in mediating IgG biodistribution and influencing rates of IgG catabolism. Consequently, ALPs possessing multiple Fc domains (Table 1) may exhibit differential FcRn binding kinetics compared to antibodies of the standard IgG format, which in turn could impact their biodistribution and pharmacokinetic profile. Similarly, ALPs lacking an Fc domain (Table 1) will not exhibit FcRn binding and therefore, their pharmacokinetics will not be influenced by that recycling system.

Differences in tissue perfusion, ratio of interstitial to vascular space, and capillary structure (continuous *vs* discontinuous) give rise to large differences in antibody concentration within the interstitial compartment of individual tissues [101,103,105,110,111], and in turn impact the distribution of therapeutic mAbs and add further complexity to the mechanism of FcγR signalling. It is estimated that anywhere between 40 and 70% of total IgG may be present in the interstitial fluid in a particular tissue [112]. Moreover, interstitial fluid is the medium through which antibodies may access membrane bound targets, therefore analysis of IgG concentration within this compartment may be relevant to the mechanism of therapeutic mAb. Furthermore, differences in interstitial concentrations across the body may have implications in disease pathology in relation to competing endogenous IgG and penetration of therapeutic mAb or ALP. The biodistribution mechanisms of mAbs has been extensively reviewed by Tabrizi et al. [111] and will not be discussed in this review.

Analysis of interstitial IgG concentrations have been conducted *via* enzyme linked immunosorbent assays (ELISA) to derive interstitial tissue concentrations and develop a more accurate PBPK model [105]. As seen previously, the half-life of mutated IgG that does not bind detectably to FcRn dropped rapidly, from 206 h to 9.87 h [105]. The results from the WT mAb indicated the lowest concentration of IgG was found in the brain, heart and liver (0.197%, 0.914% and 1.17% respectively), while most was found in the bone, spleen and skin (2%, 4.28% and 7.39% respectively). The low concentrations of IgG observed in the liver could arise due to high rates of catabolism [101,103], while a lack of IgG in brain is likely due to the presence of the blood brain barrier (BBB) [113]. When antibody concentrations were assessed in FcRn KO compared to WT mice, the biggest percentage increase was observed in the heart, adipose and liver (130.9%, 131.9 and 167.5% respectively), perhaps indicative of the necessity of FcRn for efflux from the interstitial space. In contrast, the largest reduction was observed in skin, muscle and brain (62.0%, 44.4% and 37.1% respectively). In this instance, it is hard to ascertain whether this is indicative the need for FcRn for influx into the interstitial space, or an artifact of global IgG reduction due to increased catabolism. Notably, the concentration of IgG in tumours was estimated to be at anywhere between 12 and 24% and dependent on the level of vascularisation within a given tumour, but had no dependence on FcRn for IgG distribution. Even at the lower end of this predicted concentration range, this would still place local IgG concentration above the FcγR affinity (discussed in section 4: The signal-to-noise problem in IgG signalling), which has important implications for anti-tumour mAb therapy.

An alternative PBPK model for quantifying antibodies within the interstitial fluid has been proposed by Eigenmann et al. [110]. Data extrapolated from this model suggested that interstitial IgG concentrations are reflective of plasma concentrations in tissues with discontinuous capillaries, such as the liver and spleen. In contrast, IgG in tissues with tight and size selective capillaries are restricted to the vascular space, which applies to the brain and kidney which have the blood brain barrier (BBB) and glomerular filter respectively. Finally, the remaining tissues explored in this study (lung, heart, muscle, bone, skin, gut and adipose) possess continuous capillaries, in which 50–60% of the plasma IgG concentration is found in the interstitial space.

Although the large variation in PBKK models proposed by Eigenmann et al. and Chang et al. make it difficult to ascertain the precise IgG concentrations within the interstitial space of a given tissue, they are in agreement that a large proportion of IgG is localised to the spleen. The spleen is the largest secondary lymphoid organ in the body and exhibits a wide variety of functions including clearance of red blood cells and initiation of immune responses [114,115]. Splenic macrophages may contribute to tumour removal in haematological malignancies [116], and their function may be impaired in metastatic solid cancers [117]. Notably, high concentrations of IgG in the spleen could impede phagocytosis due to FcγR saturation, therefore raising the threshold of ITAM activation. Moreover, splenic macrophages may also be involved in mediating the mechanism of action of IVIg [118], which will be discussed in section 4: The signal-to-noise problem in IgG signalling.

In contrast to the high concentration of IgG reported to occur in the spleen, it has been shown that IgG levels in the brain are negligible [105,110]. FcγRs present on microglia in the brain are thought to be unoccupied, due to the poor permeability of IgG across the blood brain barrier (BBB) [113]. This may indicate that FcγRI is more predominant in mediating effector function in the brain, although further evidence is needed to support this. Notably, the BBB is thought to be highly important for protecting the brain from brain-reactive antibodies, which may circulate in over 90% of individuals [119]. Additionally, breakdown of the BBB has been attributed to the development of Alzheimer’s disease by allowing Aβ peptides and autoreactive antibodies to enter the brain [120,121]. Moreover, further evidence suggests that the formation of ICs in the brain can lead to FcγR-mediated inflammation [122] and contribute to the pathogenesis of neurological diseases.

## 4. The signal-to-noise problem in IgG signalling

The impact of competing IgG on antibody effector function can, at one level, be understood by consideration of the binding kinetics of individual antibody receptors. The interaction between an antibody and its receptor can be understood as forming an equilibrium between the associated, free antibody and receptor bound form. This can be represented by the following expression where concentration is indicated by square parentheses.

[IgG]+[FcγR]⇌[IgG:FcγR]

Consideration of the ratio between the product of the concentrations of antibody and receptor to that of the bound form yields the equilibrium constant ($K_{\text{D}}$). Taking the approximate equilibrium $K_{\text{D}}$ for the low affinity IgG receptors as 100 nM and, for simplicity, the prevailing free serum IgG concentration as 100 μM [49], we can determine the ratio between free Fc receptor and bound IgG.

$$K_{D}=\frac{\left[\text{IgG}][\text{Fc}\gamma \text{R}\right]}{\left[\text{IgG}\;:\;\text{Fc}\gamma \text{R}\right]}$$

$$100\;\text{nM}=\frac{\left[100\;\mu \text{M}][\text{Fc}\gamma \text{R}\right]}{\left[\text{IgG}\;:\;\text{Fc}\gamma \text{R}\right]}$$

$$\frac{\left[\text{Fc}\gamma \text{R}\right]}{\left[\text{IgG}\;:\;\text{Fc}\gamma \text{R}\right]}=\frac{100\;\text{nM}}{100\;\mu \text{M}}=\frac{1}{1000}$$

Therefore, the biophysical model of this antibody receptor interaction would indicate that only 1:1000 receptors would be in a free, unbound state in the context of prevailing serum IgG. These considerations might lead to the conclusion that competing antibodies completely precluded antibody effector function, which clearly contradicts their known efficacy in the recruitment of the FcγR expressing immune cells. Although antibodies can function in the context of competing IgG, their efficacy has been demonstrated to be substantially limited in both *in vitro* assays [1,2,123–125] and *in vivo* [2,3].

The abundance of IgG within serum and interstitial space presents a unique set of challenges for FcγR signalling. Endogenous IgG is reported to circulate in human serum at 7–16 mg/mL (45–110 μM) in healthy individuals [49], which may suggest that FcγRI is fully occupied. Moreover, FcγRI is postulated to be the FcγR most impeded by saturation by endogenous IgG [126,127]. Observations that the IgG-degrading enzyme IdeS can eliminate IgG bound to cellular FcγRI [2] may improve FcγRI-mediated effector function.

FcγRI is constitutively expressed on macrophages and DCs, and can be induced on the surface of neutrophils, mast cells, and eosinophils [128]. FcγRI appears to be multifunctional, with reports that it is involved in the induction of cytokine release and inflammation [129–131] and contributes to antigen presentation in macrophage and DC subsets [132,133]. It has also been proposed to function as a scavenger receptor [134], due to the likelihood of receptor saturation at physiological concentrations of IgG. FcγRI may also be capable of mediating of ADCP in mouse macrophages [135] and ADCT in human macrophages [136,137], sometimes referred to as ‘antigen shaving’. Loss of CD20 from chronic lymphocytic leukemia (CLL) cells has been observed in patients following rituximab treatment [138,139] which may be an escape mechanism. Moreover, removal of antigen from the target cell surface associated with ADCT is thought to impede the therapeutic benefit of anti-cancer mAbs by enabling resistance [136–139]. An ADCT-mediated reduction in CD20 expression from CLL target cells has been demonstrated in *in vivo* murine models [137] and was also shown to be dependent on Fc engagement with FcγRI [136,137]. Further analysis also revealed no loss of target cell viability associated with ADCT, although this was only measured after 45 min of coculture and is contradictory to more recent evidence [140] which will be discussed below. More recently, loss of CD20 has also been attributed to a process known as modulation, whereby mAb:CD20 complexes are internalised by the tumour cells and was shown to predominate over ADCT as a mechanism of CD20 removal from the tumour surface [141]. In addition, ADCT has been demonstrated to lead to death of tumours cells following long-term coculture with macrophages [140], and has been proposed to be an important mechanism of tumour removal.

The inhibitory effect of competing IgG on FcγRI can be bypassed by using bispecfic F(ab’)_2_ fragments (Table 1) that are capable of engaging FcγRI+ effector cells with the cellular target [20]. The potential therapeutic value of FcγRI engagement is further illustrated by the development of aglycosylated antibodies that exhibit impaired interaction with low affinity receptors, and have been engineered to display enhanced FcγRI interactions [142]. Elimination of FcγRI saturation by endogenous antibody may enhance effector cell mediated cytotoxicity by monoclonal antibodies [2], as discussed below.

The low affinity FcγRs may also be occupied at serological concentrations of IgG [143], although the affinity is such that the binding between these FcγRs and monomeric IgG can often be below detection limits [144,145]. The low-to-moderate affinity FcγRs, FcγRIIA, FcγRIIB and FcγRIIIA, have two extracellular domains, and depend on Fc avidity to mediate effector function. In particular, multiple Fc-FcγR interactions may be generated at the cell surface following engagement with IC [146] or antibody opsonised target cells [147] which facilitates FcγR clustering and ITAM phosphorylation.

The most widely expressed FcγR is FcγRIIA, and is found on macrophages, DCs, basophils neutrophils, mast cells, and eosinophils. The function of this receptor is best characterised in macrophage populations and is postulated to be the predominant FcγR involved in induction of ADCP of opsonised targets [148,149], although there is evidence that FcγRI [150] and FcγRIIIA [151] also play a role. Notably, induction of ADCP has been reported to be reduced in the presence of serum IgG *in vitro* [30,152]. It is possible that high levels of monomeric IgG compete with IC for FcγRIIA engagement, thus impeding ITAM signalling. This is further supported by evidence that monomeric IgG acts as a functional antagonist of FcγRIIA by competing with IC for receptor engagement on neutrophils [143].

More recently, FcγRIIA stimulation in macrophages [140] and neutrophils [153] has also been reported to mediate ADCT. It has been proposed that FcγRIIA mediated ADCT predominates over ADCP in environments where high levels of endogenous IgG reside, with reports that the addition of serum to *in vitro* experiments with human macrophages enhances ADCT while dampening ADCP [140]. Therefore, the induction of ADCT may be more reflective of macrophage and neutrophil activity in man, with evidence that ADCT against tumour cells is an important mechanism of tumour clearance *in vivo* [140,153]. Furthermore, anti-cancer mAbs harbouring FcγRIIA enhancing mutations were found to enhance ADCT over ADCP, which contradicts reports discussed above describing no involvement of FcγRIIA. Analysis of functional activity against human immunodeficiency virus (HIV)-infected cells indicated that the main mediators of trogocytosis are FcγRIIA and FcγRIIB, although some potential involvement of FcγRI has been reported [154].

Given the different methodological approaches employed to measure ADCT in these two reports, it is possible that two distinct effector functions have been described, which are capable of occurring in conjunction with each other. One being ‘antigen acquisition’, whereby FcγRI mediated extraction of antigen from an opsonised target cell leads to its expression on the effector cell surface [136,137], potentially in a manner analogous to antigen presentation. This mechanism closely resembles classical trogocytosis as described by Joly and Hudrisier [155], which was speculated to be necessary for cell-cell communication and lymphocyte activation. The alternative effector function, coined ‘trogoptosis’ [58,153], is FcγRIIA mediated and has been shown to lead to target cell death. This mechanism may occur in a manner analogous to phagocytosis, and ultimately may be important for removal of tumours [58,153] and HIV-infected cells [154].

Given the likelihood of FcγRI saturation by endogenous IgG, the functional relevance of FcγRI-mediated ADCT *in vivo* remains to be determined. Moreover, the ramifications of endogenous IgG on FcγRIIA signalling and subsequent impact on ADCP / ADCT activity are also unclear.

High levels of serum IgG may also have functional consequences in macrophage and natural killer (NK) cell populations expressing FcγRIIIA. Engagement of FcγRIIIA with immune complexed Fc elicits antibody dependent cellular cytotoxicity (ADCC) in NK cells, which involves the release of cytotoxic granules causing target cells apoptosis in a manner analogous to T cell killing. Although typically considered an activating receptor, there are reports of an inhibitory role of FcγRIIIA following interaction with monomeric IgG. Several studies have demonstrated that pre-treatment of NK cells and macrophages with monomeric IgG preparations reduces ADCC or ADCP activity respectively, in an FcγRIIIA dependent manner [156–158]. The pathway involved in the transmission of inhibitory FcγRIIIA (iFcγRIIIA) signals remains unclear, with conflicting reports regarding the involvement of the SH2-containing tyrosine phosphatase (SHP), SHP-1 [158,159]. The possibility of SHP-1 involvement may be indicative of a signalling mechanism analogous to that in B cells, whereby continuous SHP-1 signalling has been shown to prevent BCR signal transduction [160] and may be necessary to maintain autoreactive B cells in a state of anergy [161]. Similarly, tonic FcγRIIIA signalling by endogenous serum IgG may be important for maintaining NK cells in an inactive state in healthy individuals.

**Fig. 3.**
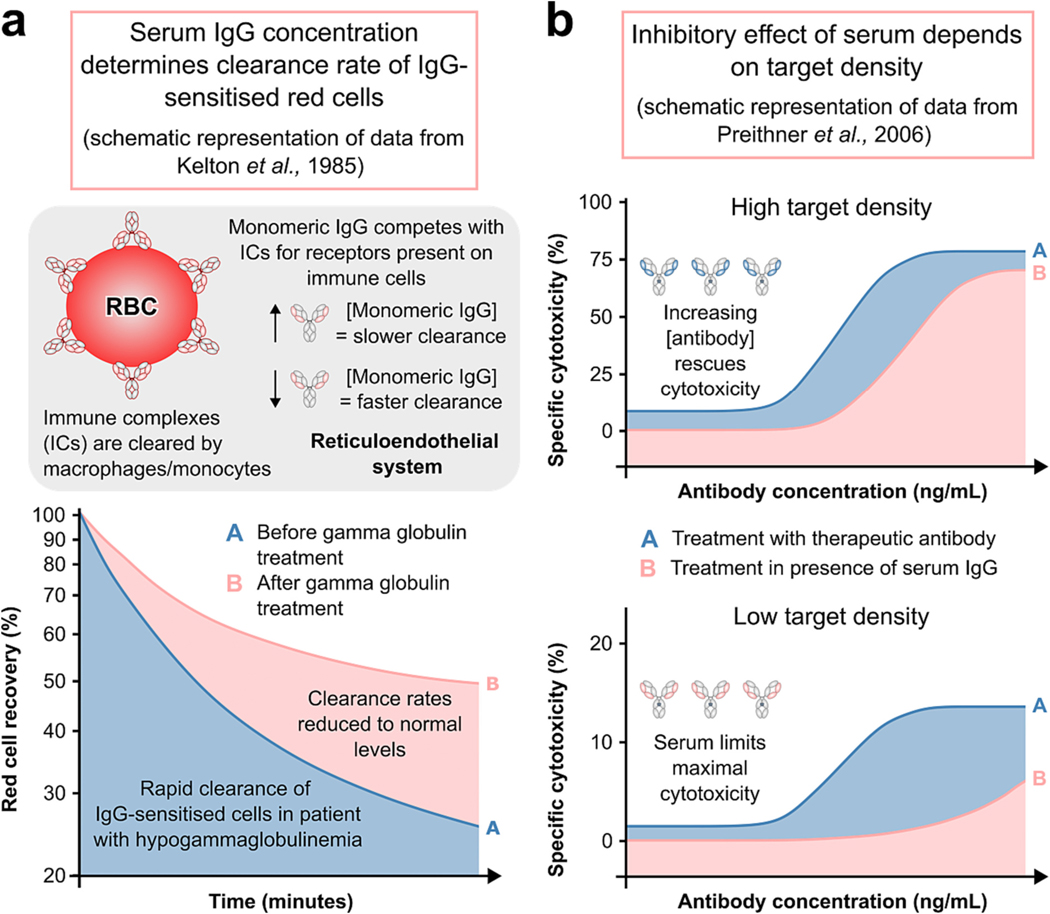
Effect of serum IgG concentration on antibody function. a Schematic representation of data from Kelton et al. (1985) [3], showing effect of serum IgG concentration on clearance rate of IgG-sensitised red blood cells (RBCs). Unusually fast clearance rate in a patient with hypogammaglobulaemia was reduced to a normal level following gamma globulin treatment. b Schematic representation of data from Preithner et al. (2006) [1], showing how the inhibitory effect of serum IgG depends on target antigen density. The inhibitory effect of competing serum IgG can be overcome using higher concentrations of therapeutic antibody when target density is high; however, the maximum antibody response is limited where target density is low, and cannot be overcome with increasing therapeutic antibody concentration. (For interpretation of the references to colour in this figure legend, the reader is referred to the web version of this article.)

The presence of competing serum IgG, along with the potential occurrence of iFcγRIIIA, may have implications for mAb-based therapeutics which are often used in the treatment of cancer. The first generation of anti-cancer mAbs were those which target the tumour cells directly by binding to surface antigens, including rituximab (anti-CD20), trastuzumab (anti-human epidermal growth factor receptor-2; HER-2) and cetuximab (anti-epidermal growth factor receptor; EGFR). Interaction with so called ‘tumour-associated antigens’ occurs *via* the mAb Fab domains and may block downstream pro-tumoral pathways, although research suggests that immune-mediated cell death may also be induced *via* engagement of the Fc domain with FcγRs on immune effectors. However, the addition of serum IgG to *in vitro* ADCC assays has been shown to reduce NK-mediated killing of tumour cells [1,162], which may be attributable to a reduction in the availability of FcγRIIIA or to inhibitory ITAM signalling. Notably, it has been shown that antibodies directed to high density targets are impacted by endogenous IgG, but that reduction in cytotoxicity can be overcome by using higher mAb concentrations [1]. However, in cases where target density is low, competing IgG had the effect of limiting maximal cytotoxicity [1] which cannot be overcome with excess mAb. This establishes competing IgG as an important parameter in the efficacy of mAb therapy requiring receptor engagement. Fig. 3 illustrates this effect, along with the impact of serum IgG on clearance of IgG-sensitised red cells demonstrated by Kelton et al. [3].

A reduction in serum IgG can be achieved by using enzymes such as the IgG-degrading enzyme of *Streptococcus pyogenes* (IdeS, also known as Imlifidase) which cleaves IgG at the hinge region. The use of IdeS has been shown to enhance the efficacy of anti-cancer mAbs in *in vitro* assays supplemented with serum antibody [163] which was attributed to a reduction in competition for FcγRIIIA occupancy, although iFcγRIIIA signalling may also be a contributing factor. IdeS has been reported to liberate receptor bound-IgG and potentiate therapeutic efficacy of rituximab in *in vivo* murine models [2]. This potentiation is achieved by models that mimic staggered administration, allowing for the rapid clearance of IdeS prior to the administration of antibody therapy. Importantly all FcγRs are liberated, including FcγRI, and therefore, it is anticipated that FcγRI+ effector cells will contribute to anti-tumour effects. Of significance, a phase II clinical trial conducted in chronic kidney disease patients found that IdeS was not only safe and well tolerated by patients, but also rapidly degraded serum IgG to *<*1%, which remained low for a 7 day period [164]. IdeS is well-tolerated in patients and has now received approval for ‘desensitisation treatment of highly sensitised kidney transplant patients with positive crossmatch against an available donor’ [165]. Analysis of serum samples from these patients also revealed that the IdeS pre-dose sera inhibited rituximab-mediated ADCP of target cells, whereas the sera collected 24 h after IdeS dosing did not block ADCP [2]. Notably, the transient effect of this treatment suggests that it could be suitable as a preconditioning regime for antibody therapy. However, repeated infusions would likely be required to sustain low IgG levels and IdeS immunogenicity could become an issue. Taken together, these findings highlight the potential of IdeS for improving anti-cancer-mAb therapeutics and this approach warrants further exploration.

The second generation of anti-cancer mAbs are those which are immunomodulatory, mediating their anti-tumoral activity either by blocking inhibitory immune signals (checkpoint inhibitors) or by stimulation of co-receptors (agonists). Checkpoint inhibitors, such as Pembrolizumab (anti-programmed cell death protein-1; PD-1) and Ipilimumab (anti-cytotoxic T-lymphocyte associated protein 4; CTLA4) bind to immune cells rather than directly to the tumour, and therefore may not require interactions with activating FcγRs. In particular, anti-PD-1 antibodies on an IgG1 background have been shown to eliminate CD8+ tumour infiltrating lymphocytes leading to abrogated therapeutic efficacy, an effect which can be overcome using Fc-null mutants that lack the ability to bind FcγRs [166]. For this reason, pembrolizumab and nivolumab (both anti-PD-1) were developed on an IgG4 background to reduce activating FcγR engagement. However, IgG4 may still bind FcγRI at a $k_{\text{a}}$ of 3.4 × 10^7^ M^−1^ [144] and may also be capable of engaging the lower affinity receptors when in IC [167]. In such circumstances, the presence of competing IgG may actually be useful for preventing IgG4 engagement with activating FcγRs, although this has not been explored.

In contrast to checkpoint inhibitors, agonistic immunomodulatory antibodies, such as anti-CD40 mAbs have been shown to require engagement with the inhibitory FcγRIIB to trigger immune activation [168,169]. CD40 is a costimulatory protein expressed by APCs which trigger immune cell activation following engagement with its ligand CD40L. Activation of CD40 expressing cells may also be achieved using agonistic anti-CD40 antibodies, which are currently been investigated in the clinic for treating a variety of malignant solid cancers [170–172]. Analysis of this mechanism has been shown to be dependent on mAb cross-linking between Fc and FcγRIIB, but not on FcγRIIB signalling [169]. As such, FcγRIIB has been postulated to act as a scaffold which enhances CD40 clustering at the membrane and subsequent downstream signalling [173]. The same may also be true of anti-41BB mAbs, which have been shown to stimulate 4–1BB on T cells to enhance their anti-tumoral effector function [174]. Notably, the agonistic ability of CD40 antibodies may be improved or impeded depending on IgG subclass [175], and should therefore be an important consideration when designing agonistic immunomodulatory antibodies. Moreover, the potential requirement for FcγRIIB engagement to promote ‘scaffolding’ indicates that the efficacy of agonistic antibodies may also be impacted by competing IgG.

In order to comprehend the signal-to-noise problem, we need to understand IgG catabolism in disease states. Increased levels of serum IgG, or hypergammaglobulinemia has been described in patients with chronic infections, including HIV [176,177] and Hepatitis C [178,179], as well as some autoimmune diseases such as rheumatoid arthritis (RA) [180,181], systemic lupus erythematosus (SLE) [182,183] and immune thrombocytopenic purpura (ITP) [184]. Patients presenting with hypergammaglobulinemia have reportedly exhibited slow Fc-dependent clearance of IC by phagocytes, while those with hypogammaglobulinemia demonstrate high clearance [3].

The occurrence of hypergammaglobulinemia associated with autoimmune disease is often attributed to the activation and expansion of autoreactive B cells [185–187], although the evidence for this is mostly limited to findings in SLE patients and murine models. Notably, hypergammaglobulinemia may be a useful marker of underlying autoimmune disease in paediatric patients [188]. In the context of HIV, the occurrence of hypergammaglobulinemia has been suggested to arise due to non-specific activation of naïve B cells by HIV-infected CD4+ T cells [189–191]. The expansion of naïve B cells is associated with a reduction in memory B cells leading to defective humoral immunity [189], and may also contribute to the increased incidence of B cell lymphomas in HIV patients [192]. The antibody levels in patients with multiple myeloma (MM) can also become extremely high [193] which may have consequences for their immune signalling thresholds. In particular, MM patients frequently experience resistance to Daratumumab (anti-CD38) [194] which could, in part, be due to competition from high levels of endogenous IgG.

The signal-to-noise problem is also a consideration when seeking to determine the mechanism of action of IVIg. Autoimmune diseases may be treated with IVIg infusions, which is produced by pooling serum IgG from thousands of healthy donors. Low dose infusions of IVIg are an effective treatment for patients with both primary and secondary immunodeficiencies as antibody ‘replacement therapy’ [195,196]. More recently, there has also been interest in the use of high dose IVIg as an ‘immunomodulatory therapy’ in HIV infected individuals. In particular, it has been reported that administration of IVIg in combination with ART reduces viremia by depletion of the latent HIV pool of CD4+ T-cells [197,198], although this phenomenon was short-lived and the precise mechanism of action is unclear.

High dose IVIg infusions have also been shown to ameliorate the symptoms of autoimmune diseases, including SLE [199], ITP [200,201] and Guillain-Barré syndrome (GBS) [202], although not RA [203,204], with conflicting evidence surrounding the mechanism by which IVIg exerts its therapeutic effects. Some have suggested that the excess IgG increases the occupancy of activating FcγRs, thus blocking their interaction with autoantibodies [205–207]. This would in turn prevent effector function activity, such as ADCC and ADCP, against autoantigens and reduce inflammation.

Notably, dimers have been shown to occur naturally in IVIg preparations [208] and may contribute to therapeutic efficacy [209–211], although their mechanism of action remains debated. It has also been postulated that sialic acid residues at the Fc glycan site, N297, are enriched within the dimer fraction of IVIg and may play a role in mediating the anti-inflammatory effects [212], which will be explored in section 5: Natural Variation. Others have demonstrated the presence of anti-idiotypic antibodies within the dimers of IVIg preparations which may block the action of autoreactive endogenous antibodies [210,211], thus providing therapeutic efficacy. The presence of dimers has also been shown to increase in the serum of SLE patients [213], which was attributed to idiotype-anti-idiotypic interactions. This phenomenon also provides rational for the formation of dimers that occur in IVIg preparations [208], which have been implicated in mediating the therapeutic efficacy of IVIg [209–211].

Perhaps the most widely accepted mechanism of action of the multimeric components of IVIg is the display of multiple Fc domains. For example, IVIg dimers show enhanced avidity to FcγRs due to their bivalent nature [209], and in theory could out compete monomeric IgG in serum. In particular, it has been demonstrated that removal of dimers from IVIg preparations abrogates therapeutic benefit in ITP mouse models [210]. However, some conflicting reports exist regarding the therapeutic impact of IgG dimers in IVIg [157], and to date, there is no definitive evidence for the hypothesis of FcγR blockade.

Although Fc receptor competition offers a compelling model for the mechanism of action of IVIg, several alternative models have also been postulated. There are reports that increased serum levels of IgG associated with IVIg infusion increases inhibitory ITAM signalling *via* FcγRIIIA which in turn reduces NK-mediated ADCC activity [157,214] and inflammatory responses [158,215]. Further supporting the notion of FcγRIIIA mediated effects are studies by Mimura et al., who found that afucosylated and galactosylated glycoforms of IVIg impeded ADCC activity with 20 times higher potency than native IgG [216]. This phenomenon was attributed to the higher FcγRIIIA affinity associated with Fc afucosylation. Moreover, it was also reported that afucosylated, galactosylated IVIg was 10 times more effective at attenuating collagen antibody induced arthritis in mice models [216].

Other studies investigating the mechanism of action of IVIg have indicated that increased FcRn occupancy enhances IgG catabolism and by default, autoantibody degradation [217,218]. Rather convincingly, it has been demonstrated that administration of IVIg with mAb increases mAb catabolism [101,219]. The same studies also found mAb catabolism was also significantly enhanced in murine FcRn KO model, and were unsusceptible to further catabolism when combined with IVIg. Alternative theories regarding the mechanism of action of IVIg have been attributed to changes in the expression pattern of the inhibitory receptor, FcγRIIB, or the glycans present on the IgG Fc domain, both of which will be discussed in the following section.

Overall, when seeking to understanding how antibodies recruit the immune system, endogenous IgG is an important parameter in defining an antibody’s signal-to-noise properties. This can be further refined by understanding the biodistribution of antibodies and how antibody concentrations change in disease states. Competition effects are also an important parameter when considering the mechanism of action of IVIg. However, the impact of competition effects can be heavily influenced by numerous variables as discussed in the following sections.

## 5. Variables influencing antibody effector functions

**Fig. 4.**
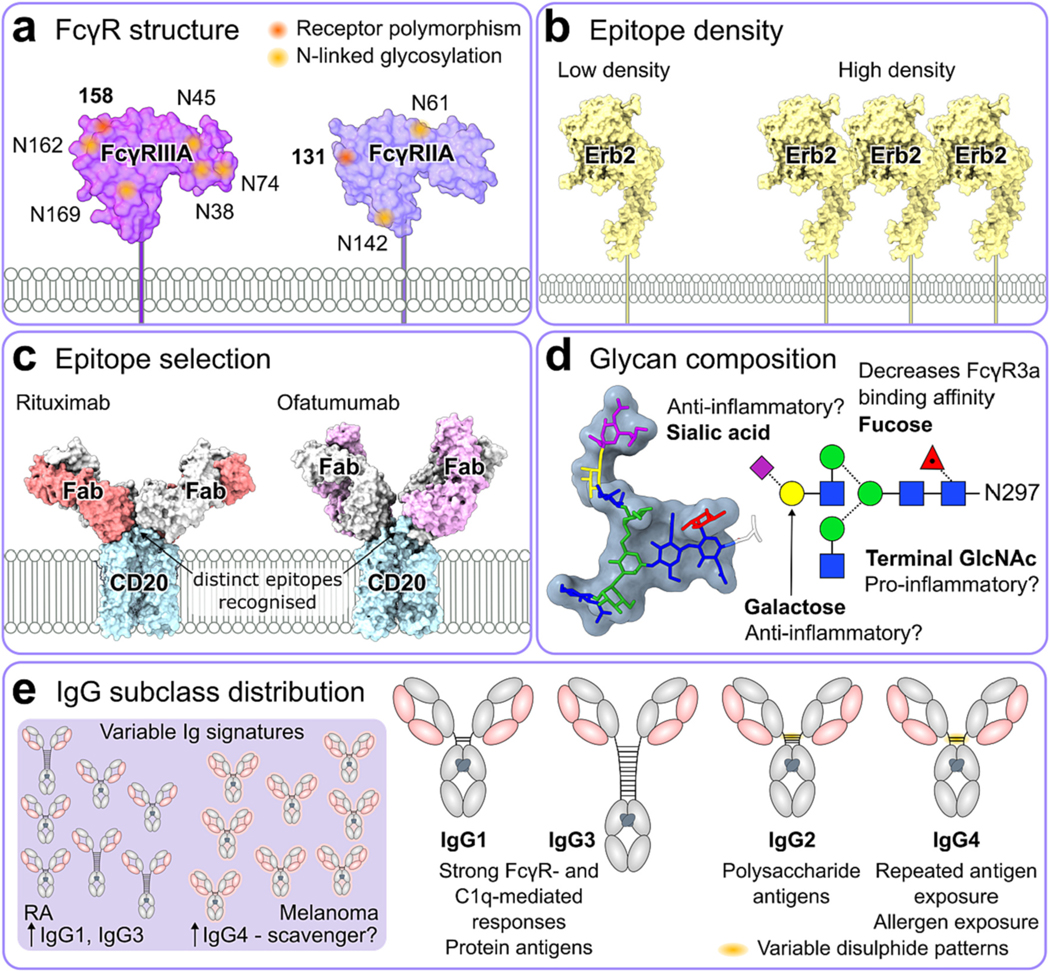
Variables influencing immune activation by IgG antibodies. a Variation in FcγR structure arises with receptor polymorphisms and heterogeneous N-linked glycosylation. Structures for FcγRIIIA and FcγRIIA are shown (PDB codes 5XJE and 3RY6, respectively). b Antigen density affects the susceptibility of an antibody to functional impediment by competing serum IgG. Antibodies targeting HER2 (PDB code 6J71) with higher affinity are less susceptible to this impediment at low antigen densities. c Distinct epitopes recognised by rituximab and ofatumumab antibodies alter their activity profile, despite both binding CD20 antigens (PDB codes 6Y90 and 6Y92 for rituximab-CD20 and ofatumumab-CD20 complexes, respectively). d Composition of Fc glycans at N297 affects affinity of IgG for FcγRs and thus influences antibody effector function. e IgG subclasses vary in their ability to engage FcγRs and stimulate immune effector functions. IgG signatures can also vary in disease states, such as rheumatoid arthritis (RA) and melanoma.

There are numerous natural variations in both antibody structure and cellular receptors that influence the effects of endogenous IgG on signalling thresholds (summarised in Fig. 4). Understanding these has the potential to help guide the development of enhanced therapeutic antibodies, discussed in section 7.

### 5.1. Epitope selection

The F(ab’)_2_ variable region dictates the epitope specificity of a given IgG and for many years has been thought to play no role in antibody effector function. However, recent research into anti-cancer mAbs has suggested that the distance of the epitope from the tumour cell surface can influence the potency of ADCC and ADCP [220]. Specifically, epitopes proximal to the cell membrane were found to favour ADCC, while distal epitopes favoured ADCP. This finding provides rational as to why ofatumumab, a mAb binding to the membrane proximal region of CD20, can more effectively mediate ADCC compared to rituximab [221,222] (Fig. 4c).

In addition, a mAb cocktail targeting multiple HER2 epitopes was found to mediate more potent ADCC responses against the human breast cancer cell line BT474 compared to trastuzumab alone [223]. Although the reasons for this were not explored, this may be attributable to a greater Fc density at the cell surface, or alternatively, a broader range of Fc orientations available for FcγRIIIA engagement. Cocktails of anti-HER2 mAbs have also demonstrated reduced tumour growth *in vivo*, which was attributed to increased HER2 endocytosis [224]. However, loss of HER2 from the tumour surface would likely reduce immunological mechanisms of action such as ADCC and ADCP. It is therefore possible that trogocytosis between HER2 expressing targets and macrophage or monocyte populations also played a role in impeding tumour growth *in vivo.* The sensitivity of mAb-mediated ADCC and ADCP to suppression by endogenous IgG [1,162] means that targeting multiple epitopes could be a useful way of improving such responses *in vivo*.

The angle at which mAbs bind their epitopes has also been implicated in the effector function potency; mAbs targeting overlapping epitopes on the HIV-1 envelope glycoprotein, gp120, can exhibit up to 75-fold difference in ADCC potency which was found to be independent of antigen binding affinity and associated with variation in antibody orientation when bound to antigen [225]. Such findings may be important for informing the decision of epitope selection in future mAb development and again, may also be a useful tool for overcoming competition for FcγRIIIA engagement by serum IgG.

### 5.2. Epitope density

There is evidence that IgGs are mobile when bound to cell surface antigen targets and exhibit ‘bipedal’ stochastic walking [226]. This mobility may enable bivalent epitope binding [227], as well as facilitate Fc clustering and serve as a docking site for FcγR interactions. However, the ability of an antibody to form bivalent interactions is in part influenced by the density of its target. In particular, it has been reported that higher epitope densities increase the proportion of bivalent interactions over monovalent interactions between antibody and its target, which is also associated with a slower rate of dissociation [228].

The consequences of antigen density have been explored in the context of HIV, for which it was reported that HIV-1 neutralising antibodies with low binding affinity to gp120 are sensitive to variation in antigen density, and are associated with a reduction in antigen recognition at low epitope density [229]. Moreover, the observed low density of gp120 on the viral surface [230–232] is likely to compound this issue further by limiting the occurrence of bivalent antibody binding, which in turn may reduce viral neutralisation [233].

The relationship between epitope affinity and antigen density has also been explored in the context of anti-cancer therapeutics. Antibody clones targeting epithelial cell adhesion molecule (Ep-CAM) or HER2 with high affinity have been demonstrated to be less susceptible to functional impediment at low antigen densities [234,235]. Such findings provide rationale for the need to design therapeutic anti-cancer mAbs with high epitope affinity to reduce the influence of antigen density on efficacy. Notably, mAbs which bind tumour surface antigens exhibiting high densities are associated with more potent ADCC responses [234,235], while other studies have reported a reduction in NK-mediated ADCC associated with low levels of target antigen expression [236]. Such findings have been attributed to lower levels of antibody coating the target, and implies Fc aggregation at the cell surface is necessary for enabling FcγRIIIA engagement.

Low epitope density may also be differentially impacted by serum IgG [1] (Fig. 3b). Preithner et al. showed that the maximal ADCC activity of adecatumumab (anti-Ep-CAM) and trastuzumab (anti-HER2) to high density targets in the presence of IgG required elevated mAb concentration. In contrast, elevated mAb concentration could not fully overcome the competition effects of serum IgG when target density was low [1].

Irrespective of whether the inhibitory effect of IgG on NK activity is attributable to tonic monomeric signalling or competitive FcγRIIIA engagement [156–158], it is feasible that high levels of IgG would further exacerbate the diminished ADCC activity associated with low density antigen targets. Since FcγRIIA binds with even lower affinity to IgG than FcγRIIIA, the same rationale may also be applied to ADCP. Indeed, ADCP induction is also reduced in the presence of serum [30,152], and therefore it is possible that ADCP activity is abolished at low target epitope densities *in vivo*. If this were indeed the case, would this enable preferential selection of ADCT over ADCP as a mechanism of target removal in settings where epitope density is low, or would ADCT activity too be inhibited? Many questions around this subject remain to be answered.

### 5.3. IgG subclass distribution

The different IgG subclasses exhibit variation in their ability to engage FcγRs and hence stimulate immune effector functions (Fig. 4e). Therefore, changes in their abundance within the serum has been implicated in the progression of disease pathogenesis. During infection, the abundance of each subclass may increase in response to different types of antigen [237]. For example, IgG1 and IgG3 are most often produced in response to protein antigens, whereas responses to bacterial capsular polysaccharide antigens are generally restricted to IgG2 [238,239] (Fig. 4e). Polysaccharides in particular are known to be a T cell independent type antigen and so do not rely on T cells to induce class switching to the IgG2 subclass [240], owing to their inability to associate with MHC class II molecules on the surface of APCs [241]. In contrast, the induction of IgG4 antibodies tends to predominate following exposure to allergens [242] or after prolonged antigen exposure following repeated immunisation [243] (Fig. 4e). Research suggests that the presence of IgG4 antibodies is protective against IgE mediated pathogenesis associated with allergies [243–245].

An increase in circulating IgG1 and IgG3 has been reported in RA [246], SLE [247] and GBS patients [248] (Fig. 4e). Interestingly, the isotype distribution of self-reactive antibody levels against type II collagen differ for RA and SLE, with the former being skewed towards IgG1 and IgG3 compared to a predominantly IgG4 response for the latter [249]. Given that IgG1 and IgG3 are the two subclasses which mediate the most potent FcγR-mediated effector function and complement fixation, it is possible that such collagen-specific mAbs are responsible for the inflammation and subsequent joint pain associated with RA. However, a separate study has described elevated levels of IgG4 antibodies in some RA patients [250], which has been linked to higher disease activity [251]. The potential involvement of IgG4 in the pathogenesis of inflammatory diseases such as SLE and RA is somewhat unexpected, as IgG4 antibodies are poor fixers of complement and bind weakly to FcγRs. This has led some to the conclusion that the induction of IgG4 responses is actually a protective response to block IgG1/IgG3 autoantibody engagement with self-antigen, thus damping pathogenic inflammation in SLE [252]. The ability of IgG4 to sequester antigen from IgG1 and IgG3 may also provide rational for the acquisition of IgG4 allergen titres and the induction of immune tolerance following allergen immunotherapy [243–245].

The role of IgG4 in mediating immune tolerance has also been implicated in the development of malignancies, with reports of elevated IgG4 serum levels in melanoma [253–255], pancreatic cancer [256], and glioblastoma [257]. In the context of melanoma, the presence of IgG4 was associated with secretion of T helper cell 2 (Th2)-type cytokines [253], that often occur following repeated antigen exposure and chronic inflammation [258]. In some studies, IgG4 antibodies within the tumour microenvironment were also found to be tumour specific [253,257], and may therefore compete with IgG1 antibodies for epitope binding leading to a reduction in ADCC and ADCP activity against tumoural cells.

Differences in IgG subclass may also be important in controlling the spread of viral infections. For example, HIV-1 infected individuals with stable disease have significantly higher titres of IgG1 anti-gp120 antibodies, compared to chronic progressors [259]. A separate study by Sadanand et al., also found that HIV-1 disease progressors also tended to acquire envelope-specific IgG2 antibodies, which was also associated with a loss of envelope-specific IgG3 antibodies [260]. The fact that the IgG3 isotype most strongly binds C1q [261–263] also supports the finding that complement dependent cytotoxicity is an important mechanism in response to HIV-1 infection. Furthermore, an assessment of ADCP and ADCC activity between progressors and non-progressors found no differences between the groups, although antibody-dependent cellular viral inhibition (ADCVI) was found to decline in progressors [260]. Such findings are in agreement with Richardson et al. [154], which reported that ADCT and CDC, but not ADCC or ADCP, were important in the antibody response to HIV infection. We note in the context of sterilising immunity, antibody effector functions do not contribute to protection from viral challenge [264].

### 5.4. Fc glycan structures

The activity of serum IgG and mAbs may be influenced by the composition of glycans present on their Fc glycosylation site, which in turn can impact disease pathogenesis and therapeutic efficacy respectively (Fig. 4d). The site is conserved to N297, providing stability to the Cμ2 domain and was previously thought to be essential for allowing binding between Fc and the low affinity FcγRs. However, more recent evidence suggests that aglycosylated IgG in immune complex may still bind the low affinity FcγRs, albeit at a much lower affinity than their glycosylated counterparts [167]. Although large heterogeneity is exhibited between glycans at N297 the glycan core remains constant, comprising of two GlcNAc, three mannose. Antibody glycosylation is dominated by so called complex-type glycans, where additional fucose, galactose, and terminal sialic acid residues are also added to the final structure with varying abundance and which have the capacity to influence FcγR affinity and engagement [212,265–272].

In particular, removal of fucose from the core glycan structure is known to enhance affinity between IgG1 and FcγRIIIA and increase NK-mediated ADCC activity [265–268] (Fig. 4d). The afucosylated mAb Mogamulizumab and the low fucosylated mAb Obinutuzumab, which target CCR4 and CD20 respectively, exhibit enhanced ADCC and have both received clinical approval [273–276]. Afucosylation reduced the steric hindrance between the Fc and the glycan at Asn 162 on FcγRIIIA, thereby enhancing the affinity [277,278]. The enhanced affinity FcγRIIIA associated with afucosylated glycoforms has also been demonstrated to reduce serum competition effects [125].

Interestingly, an increase in the abundance of fucosylated IgG glycans is observed following repeated immunisation [279], which may be an important mechanism for regulating NK cytotoxic activity during infection. This is further supported by the finding that afucosylated IgG titres correlates with disease severity in COVID-19 patients [280,281]. In particular, the occurrence of afucosylated IgG Fc against the SARS-CoV-2 spike protein was found to be higher in patients experiencing acute respiratory distress syndrome [280] and associated with increased levels of hospitalisation [281]. These severe responses were also attributed to enhanced FcγRIIIA affinity associated with afucosylated glycans and increased inflammatory cytokine production [281]. Interestingly, it was postulated that treatments with convalescent plasma enriched in fucosylated anti-COVID-19 antibodies could be a useful way to outcompete afucosylated anti-SARS-CoV2 IgG-responses developing in the patients [280].

Aside from afucosylation, there is also evidence that terminal galactose residues may also enhance ADCC activity [269], although not to the same extent as fucose removal [282]. However, this finding has been questioned [283,284]. More recent evidence suggests that presence of terminal galactose residues enhance the ADCC activity of afucosylated glycan structures, but have no impact when present on fucosylated glycans [270]. In contrast, there are reports that that the occurrence of terminal galactose residue positively correlates with ADCP activity in monocyte cell-line THP-1 cells [285], although interestingly this was not associated with enhanced FcγRIIA or FcγRIIIA affinity. This may indicate that FcγRI is the predominant mediator of ADCP in monocytes, although binding to FcγRI was not explored in that study.

The abundance of terminal galactose residues present on the IgG of healthy individuals [286] and the reported enhanced affinity for FcγRIIB [287] has led many to postulate that they have anti-inflammatory activity (Fig. 4d), which could be important for maintaining immune cell populations in an inhibitory state. This hypothesis is supported by the occurrence of galactose-deficient IgG Fc glycans in various diseases, including HIV [176], RA [288–290] and SLE [291,292]. Although the mechanism for this reduction in galactose is not well understood, it is possible that accelerated IgG synthesis and subsequent hypergammaglobulinemia may give rise to immature Fc glycan structures with exposed GlcNAc residues. Terminal GlcNAc residues are often considered proinflammatory (Fig. 4d) as they have been shown to fix complement *via* interaction with C1q [293], and also increase IgG uptake *via* the mannose receptor on macrophages and dendritic cells [294], which may contribute to disease pathogenesis in RA and SLE. The proposal that terminal GlcNAc can activate complement by mannose binding protein [295] has been questioned by the observation that the efficacy of antibodies with deferential galactose levels is unaffected in mice by the knockout of the mannose binding protein gene [296].

Patients with RA are also reported to have higher levels of IgG lacking sialic acid residues, particularly in autoantibody populations [288–290]. In contrast to glycan structures exhibiting terminal galactose or lacking fucose residues, terminally sialylated glycans have previously been shown to be anti-inflammatory (Fig. 4d), associated with a reduction in affinity to activating FcγRIIIA and reduced ADCC activity [212,271,272]. This impact on ADCC has been observed when core fucosylation is present, but displayed minimal influence in the context of afucosylation [297]. Studies have also demonstrated that the infusion of sialylated IgG autoantibodies in lupus nephritis and RA mouse models inhibits autoimmune pathology [298]. However, whether terminal sialic acid residues are truly anti-inflammatory is often disputed. Since most studies use sialidase treated antibodies for comparison, it is possible that differences in glycoform activity occur due to the exposure of inflammatory galactose (or GlcNAc) residues in control groups, rather than presence of terminal sialic acid residues. This is supported by a study by Thomann et al., who reported no differences in FcγRIIIA affinity and ADCC activity of IgG fractions enriched for sialylated glycans, compared to standard/native IgG fractions [269]. Interestingly, the same study also observed an enhancement of FcγRIIA binding associated with sialylated glycan structures, although the impact on ADCP or ADCT was not reported.

High levels of sialic acid residues in IVIg preparations has previously been proposed to mediate the therapeutic benefits of IVIg [299–302], although this is often attributed to engagement with SIGNR1 [301,302], a mouse gene homologous to human DC-SIGN [303]. However, other studies have disputed these findings [209,216,304], meaning the contribution of sialic acid to IVIg therapeutic activity remains uncertain. Moreover, there seems to be a lack of biophysical evidence that the Fc domain interacts with DC-SIGN regardless of sialylation status [305–307]. Nonetheless, sialylated Fc domains and antibodies are being explored in anti-inflammatory applications [308].

As mentioned previously, afucosylated, galactosylted glycoforms within IVIg preparations have been shown to inhibit ADCC activity more potently than native IVIg [216]. This may indicate that these glycoforms within IVIg preparations may particularly contribute to the attenuation of symptoms of autoimmunity.

### 5.5. FcγR polymorphisms and heterogeneity

Several FcγR single nucleotide polymorphisms (SNP) have been shown to influence affinity to IgG Fc [144], which in turn may impact the induction of antibody effector function (Fig. 4a). Those most described in the literature include *FCGR2A H131R, FCGR2B I232T* and *FCGR3A F158V*, and these have been implicated in predicating disease progression and response to antibody therapeutics. In particular, *FCGR3A F158V* has been demonstrated to influence the induction of ADCC *in vitro* [236], with further clinical data suggesting that patients homozygous for the lower affinity allele *FCGR3A-F158* have reduced progression free survival (PFS) following therapeutic mAb treatment compared to those who are *FCGR3A-V158* homozygous or heterozygous [309–314]. This could be indicative of the importance of ADCC in mediating tumoral cell removal and suggests that affinity between Fc and receptor influences effector function potency. However, many of these studies are small and underpowered, and the 99% sequence homology between *FCR3A* and *FCR3B* means discriminating between the two could also influence outcomes [315]. Furthermore, more recent large-scale studies describing no involvement of FcγR SNPs in therapeutic responses to anti-cancer mAb therapy have also been published, implying that *FCGR3A-F158* may not be as detrimental to PFS as originally thought [316–321].

There is also uncertainty regarding the influence of *FCGR2A H131R* and *FCGR2B T232* in anti-cancer therapy. Differences in IgG1 affinity to *FCGR2A- H131 and FCGR2A- R131* are negligible [144] and therefore may lack functional consequence, however, they have still been compared in response to anti-cancer mAb treatment. While *FCGR2B I232T* polymorphisms do not alter the affinity between receptor and Fc [322], acquisition of *FCGR2B- T232* has been shown to result in exclusion of FcγRIIB from activating receptor lipid rafts and is associated with a loss of inhibitory FcγRIIB signalling [322,323]. Hence, both *FCGR2A H131R* and *FCGR2B I232T* polymorphisms could impact mAb mediated ADCP or ADCT against tumoral cells, although as for *FCGR3A F158V*, many conflicting results have been reported [309,310,312–314,317–319,321,324,325] which may be attributable to sample size or SNP detection technique [315]. Inconsistencies in the significance of these SNPs could be due to the influence of other patient factors, such as cancer type, treatment regime and disease stage, which make it difficult to ascertain whether there is truly an association between FcγR polymorphisms and response to anti-cancer mAb therapy.

Notably, there are reports of significant association between SLE susceptibility and *FCGR2B- T232* acquisition in Asian populations [326,327], which may be attributable to a lack of inhibitory signalling in B cells [322] and macrophages [323]. The same studies [326,327], along with others [328–331], have also implicated the inheritance of *FCGR3A-F158* in SLE, which is somewhat unexpected given the apparent increase in ADCC potency associated with this polymorphism, as demonstrated *in vitro* [236]. This could be indicative of reduced iFcγRIIIA-mediated signalling by monomeric IgG in *FCGR3A-F158* individuals, resulting in a loss of NK anergy and associated clinical implications. However, this is speculative as there is a lack of evidence indicating an association between FcγRIIIA polymorphisms and their impact on tonic signalling.

In addition to polymorphisms, FcγRs are also structurally heterogenous due to differential processing of their N-linked glycans. Glycosylation has been shown to influence the threshold of FcγR-mediated immune activation and the relationship between cell type activation status and receptor glycosylation is an area of active research [332].

Overall, numerous variables influence antibody effector functions. A recent study has demonstrated that C1q concentration and other serum factors can also infleunce ADCC underscoring how antbody effector functions are infleunced by the complex local environment [333].

## 6. The inhibitory nature of FcγRIIB

In contrast to the activating receptors, FcγRIIB signals *via* a cytoplasmic immunoreceptor tyrosine-based inhibitory motif (ITIM), which abrogates ITAM signalling. Specifically, aggregation of ITIMs recruit SHP-1 [334] and the SH2 domain containing inositol phosphatase (SHIP) SHIP-1 [201], which have been shown to inhibit tyrosine phosphorylation of Syk kinase [335] and dephosphorylation of phosphatidylinositol triphosphate (PIP3) respectively [336]. Stimulation of FcγRIIB on macrophage populations inhibits the phagocytic activity of FcγRIIA [335,337], which may be important in regulating the immune response during infection [338].

Beyond its inhibitory function, there is evidence of FcγRIIB involvement in mAb internalisation [98,339–341], IC clearance [342] and trogocytosis [154,343]. In particular, recent evidence suggests that mFcγRII-mediated IgG internalisation is in part responsible for the rapid clearance of mAb in non-obese diabetic (NOD) severe combined immunodeficient mice (SCID) mice, which was associated with reduced mAb efficacy compared to that seen in SCID mice [98]. It was proposed that a reduction in FcRn expression associated with NOD SCID mice prevented IgG salvage from mFcγRII in the lysosome, leading to enhanced mAb degradation [98]. While the absence of endogenous IgG exhibited by NOD SCID mice was reported to not be a contributing factor in the altered mAb pharmacokinetic profile, reconstituting mice with mIgG to levels comparable with BALB/c mice prevented rapid mAb clearance. Notably, the expression of mFcγRII on liver sinusoidal endothelial cells (LSECs) has been reported to account for approximately three quarters of the total mFcγRII in mice [342]. Given previous reports that the liver is the major site of IgG catabolism [101,103], it is possible that the high expression levels of mFcγRII on LSECs contributes to high rates of IgG internalisation and degradation. However, it remains to be established whether FcγRIIB plays a similar role in humans.

The expression of FcγRIIB by malignant B cells has also been demonstrated to mediate internalisation of CD20:mAb complexes on the cell surface [339,340,344] which may reduce clinical efficacy of anti-CD20 therapeutics. The expression pattern of FcγRIIB has also been implicated in the development and procession of other cancer types, with reports of FcγRIIB upregulation on immune cell populations in both solid [345] and haematological [346] tumours. Moreover, recent evidence has indicated that the mechanism of reduced mAb efficacy associated with FcγRIIB is independent of ITAM signalling, and instead due to competition with FcγRIIA for Fc engagement [45]. As such, the interest in anti-FcγRIIB mAbs as a means of checkpoint inhibition has grown in recent years, with evidence that blockade of FcγRIIB may overcome immunological resistance [346,347].

There is evidence that FcγRIIB may also be capable of mediating ADCT. In particular, it has been reported that macrophage trogocytic activity is still detectable in FcR γ-chain deficient mouse models, which was also shown to occur independently of FcγRIIB-mediated internalisation of CD20:mAb complexes [343]. Like their human homologues, murine activating receptors FcγRI, FcγRIII and FcγRIV require γ-chain association to initiate ITAM signalling, while the murine inhibitory receptor FcγRII signals *via* its cytoplasmic ITIM domain. *In vitro* models using human cell lines also found that blockade of both FcγRIIA and FcγRIIB resulted in significantly reduced ADCT activity against HIV-infected cells [154]. Given that mice do not possess the FcγRIIA receptor, it is possible that the inhibitory FcγRII is the sole mediator of trogocytosis in murine models, whereas both FcγRIIA and FcγRIIB are capable of mediating this effector function in humans. Furthermore, the potency of individual FcγRIIA and FcγRIIB blockade [154] suggests that the two receptors may depend on each other to mediate trogocytosis, although there is currently no evidence to support this theory.

There are also reports that FcγRII expressed in the liver sinusoidal endothelial cells (LSECs) of mice plays a role in the clearance of IC *via* endocytosis [342], which has also been replicated in FcγRIIB expressing LSECs in rat models [348]. It has also been demonstrated that truncation of the FcγRIIB cytoplasmic domain in BHK-21 cells leads to loss of endocytic activity of IC [349], indicating the necessity of ITIM signalling in mediating this mechanism. However, more recent evidence has shown that ITIM signalling is not required for endocytosis of rituximab-ligated CD20 from the surface of Ramos cells [350]. This may be reflective of the differential mechanisms of internalisation between cis interactions between FcγRIIB and Fc on the same cell surface, compared trans interactions in which FcγRIIB binds with IC in solution. Notably, the robust inflammatory responses observed in FcγRII−/− murine models stimulated with IC [351] has led to the hypothesis that FcγRIIB/FcγRII plays a role in the development of autoimmunity and is important for mediating the tolerogenic properties of LSECs [7,352].

Clinical studies have observed down-regulation of FcγRIIB on DCs in SLE patients [353] and upregulation of FcγRIIB expression on DCs in RA patients with low disease activity [354], suggestive of a protective role against pathogenic inflammatory responses. There is also evidence that FcγRII expressing macrophages reduce joint inflammation in murine models by promoting endocytosis and clearance of ICs [355]. This, combined with the observed IC deposition within the joints of RA patients [355], suggests that impaired FcγRIIB-mediated IC removal may contribute to the pathogenesis of RA, and potentially other autoimmune diseases.

Further evidence of macrophage involvement has come from studies in FcγR-humanized mice which have demonstrated upregulation of FcγRIIB in splenic macrophages following IVIg treatment *in vivo* [118]. This finding is supported by studies that have shown that FcγRII^−/−^ ITP murine models lack therapeutic benefit associated with IVIg treatment [299], although it has been reported that this finding is strain specific [356]. Upregulation of FcγRIIB has also not been observed following IVIg treatment of human monocytes and macrophages isolated from peripheral blood of healthy donors [209], nor from patients with Kawasaki disease [357] or ITP [358], despite the fact that Kawasaki disease and ITP patients typically benefit from IVIg treatment. The discrepancies between the studies could be indicative of differing IVIg responses between splenic and peripheral monocyte/macrophage populations or be reflective of the variation in FcγRII/FcγRIIB signalling in mice and humans.

The therapeutic mechanism of IVIg has also been attributed to B cells, with evidence from SLE patients that IVIg treatment can prevent B cell activation [359]. This, combined with evidence of B cell anergy [360] and inhibition of antigen presentation following IVIg treatment [361] suggests that they, rather than innate cells, could be the dominant cell types in mediating therapeutic benefit associated with IVIg. Of significance, FcγRIIB expression in B cell populations is known to regulate BCR signalling and is dependent on SHIP recruitment [362–364]. A reduction in FcγRIIB expression level has been observed in memory B cell populations in patients with SLE [365] and RA [366] which could be indicative of a loss of FcγRIIB-mediated checkpoint inhibition and enabling the expansion of autoreactive B cells. Additionally, studies have also shown that FcγRII^−/−^ murine models develop self-reactive germinal centre B cells [367], which may provide rationale for the down-regulation of FcγRII observed in germinal centre B cells in *in vivo* SLE models [183].

## 7. Engineering solutions

**Fig. 5.**
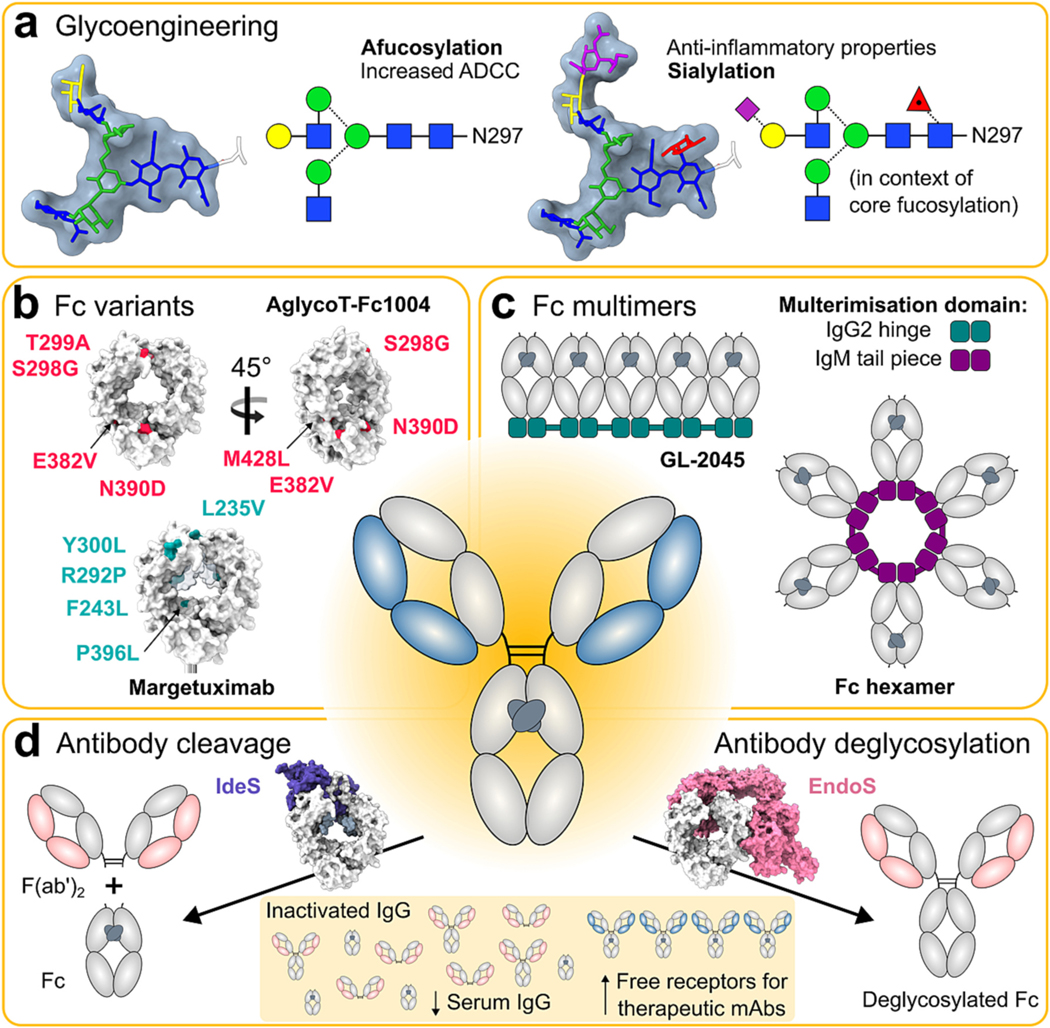
Engineering solutions for improved antibody therapeutics. a Glycoengineering of Fc N-linked glycans can be used to improve antibody function, such as by removal of core fucose to increase affinity for FcγR3a. b Specific mutations can increase antibody function and/or confer selectivity towards activation of a particular receptor. Mutations present on anti-HER2 antibody AglycoT-Fc1004 and mutations enhancing FcγR3a affinity (being utilised in Margetuximab development) are mapped onto a wild-type IgG1 Fc structure (PDB code 3AVE). c Strategies for Fc multimerisation using IgG2 hinge and IgM tail piece domains, which can possess increased engagement with FcγRs due to their higher avidity. d Strategies for inactivation of serum IgG using streptococcal IgG-degrading enzymes IdeS (PDB code 8A47) and EndoS (PDB code 8A49), which specifically cleave and deglycosylate IgG Fc, respectively. Such biologics could be particularly useful in tandem with therapeutic antibodies designed to be resistant to inactivation [163] or administered after enzyme clearance [2,368].

While Fc-dependent antibody therapeutics represent a significant advancement in the field of medicine and are now widely employed for treating a wide array of pathologies, more recent research has focused on further improving upon these therapeutics by means of Fc engineering (summarised in Fig. 5). Indeed, various Fc modifications have been explored to either impede or enhance engagement with FcγRs, depending on therapeutic application, which in turn may improve efficacy by overcoming competition from endogenous IgG.

### 7.1. Antibody fragments and single domains antibodies

As mentioned previously, bispecific antibody fragments targeting FcγRI and MHC II leads to potent tumour depletion in murine models [20] which could be an attractive therapeutic option for overcoming FcγRI saturation *in vivo*. Bispecific antibody fragments that bypass the requirement for Fc engagement with FcγRIIIA to stimulate an immune response have demonstrated some clinical success [21,22]. In particular, AFM13 (previously TanAb) is a tetravalent bispecific CD30/FcγRIIIA tandem diabody consisting solely of Fv domains and leads to potent lysis of CD30+ targets [21]. Moreover, AFM13 was found to not induce lysis in an unspecific manner to CD30- targets, and remained bound to NK cells isolated from human donors in the presence of physiological concentrations of serum IgG [21]. Similarly, bispecific antibody fragments targeting CD33 and FcγRIIIA have also demonstrated potent cytotoxic responses against the Raji cells line [369]. An alternative trispecific format containing an IL-15 linker was found to exhibit higher levels of cytotoxicity compared to its IL-15-null counterpart, and was also associated with robust NK cell expansion *in vivo* [22]. Notably, the lack of Fc domain associated with the single domain antibody architecture may results in a loss of binding to FcRn, which could impede salvage from the lysosome following internalisation. Although *in vivo* half-life has not been reported in these studies, consideration should be given to ensure antibody fragment therapeutics do not experience rapid clearance *in vivo.*

### 7.2. Glycan-optimised mAbs

As mentioned previously, the composition of Fc glycans can impact antibody effector function, which can be used to the advantage of mAb based therapeutics (Fig. 5a). Examples of clinically approved ‘glyco-engineered’ therapeutics are afucosylated anti-cancer mAbs which exhibit enhanced affinity to FcγRIIIA and increased ADCC activity *in vitro* [89]. Recent evidence suggests that the increased ADCC activity associated with afucosylated mAbs is a result of enhanced shedding of the extracellular domain of FcγRIIIA from the NK cell surface, which in turn promotes ‘serial killing’ [370]. Of significance, these afucosylated mAbs were also able to overcome the inhibitory effects of serum IgG [371]. Examples of such glyco-optimised mAbs for the treatment of malignancies include Obinutuzumab (anti-CD20) and TrasGEX (anti-HER2), which have both exhibited anti-tumour activity in mouse models [26,372]. Significantly, Obinutuzumab has also demonstrated considerable benefit to PFS compared to rituximab in the treatment of CLL [373] and follicular lymphoma [374], and has therefore been approved by the United States Food and Drug Administration (FDA) as frontline and secondline treatment respectively. TrasGEX has so far completed two Phase I clinical trials, and was found to be well tolerated and exhibit anti-tumour activity [25,375]. Importantly, combinations of glycan engineering and Fc mutation that result in enhanced FcγRIIIA affinity have revealed that there is an affinity threshold which achieves maximal ADCC. Masuda et al. demonstrated that either Fc mutations or afusocylation had similar enhancement of ADCC, which could not be further enhanced by combining these engineering approaches [376]. The capacity to generate enhanced Fc through both mutation and glycan engineering has been extensively reviewed elsewhere [377–379].

The therapeutic utility of sialylated autoantibodies has also been proposed as a treatment option for autoimmune disease. Bartsch et al. [298], discussed in the previous section, found that the infusion of sialylated autoantibodies, specifically murine anti- type II collagen, attenuated disease progression in lupus nephritis and RA models. Furthermore, the necessity of antigen specificity for mediating such effects was confirmed using non-specific sialylated antibody controls. This was speculated to be due to the need for IC formation between antibody and antigen, although this does not rule out the notion of antibody sequestration from pathogenic, non-sialylated autoantigens. Ultimately, the results suggest that antigen sequestering, along with sialylated Fc glycans, are needed for the therapeutic efficacy mediated by the sialylated autoantigens and has the potential to be explored as a treatment option for autoimmune patients. Moreover, Fc mutations, such as those to the hinge glycan site Asn-221, are reported to result in poor inhibition of hemagglutination by influenza virus when expressed in HEK 293-F cells compared to CHO-K1 cells, which was speculated to be due increased sialyation at this site [380]. This work highlights the differential Fc glycosylation patterns that can emerge depending on the cell line in which antibodies are expressed, and also the functional consequence on therapeutic application.

### 7.3. Fc-optimised mAbs

Specific Fc mutants have been described that selectively enhance mAb affinity for FcγRIIA or FcγRIIIA [381], and have demonstrated superior ADCP and ADCC potency respectively *in vitro* [382,383] (Fig. 5b). Of significance, an anti-HER2 mutant (S298G, T299A, N390D, E382V, M428L) known as AglycoT-Fc1004 was not only found to exhibit superior ADCP activity compared to trastuzumab, but was also found to enhance phagocytosis against HER2 low expressing cell lines [383]. If translated further, this could be an effective treatment for patients with HER2 low tumours who reportedly do not benefit from trastuzumab treatment [384]. Moreover, the FcγRIIIA affinity enhanced mutant (featuring L235V, F243L, R292P, Y300L, and P396L) has been adopted for the development of Margetuximab, recently demonstrated superior PFS compared to trastuzumab in the SOPHIA trial (NCT02492711) [385] and is now FDA approved for the treatment of metastatic HER2 positive breast cancers. Notably, analysis of the functional activity of AglycoT-Fc1004 or Margetuximab in the presence of competing IgG has not been reported. Nonetheless, Fc mutants exhibiting enhanced affinity to activating FcγRs may be an attractive strategy for overcoming competition effects associated with serum IgG.

### 7.4. Fc multimers

Multimerization of Fc domains has been proposed to increase interaction between Fc and FcγR and can be achieved through engineering stable oligomeric forms (Fig. 5c).

Some of the of the first described Fc multimer formats include Stradomers, which were manufactured by Gliknik. These fusion proteins were based on a murine IgG2a background and were designed to mimic the dimeric fraction in IVIg. They lacked variable domains to prevent potentially damaging epitope binding *in vivo*, and instead featured a multimerization domain which allowed Fc dimerization. Purification of these structures *via* size exclusion indicated multimers of varying sizes were present in the formulation [386], potentially indicative of laddering which could be problematic for developability. Nonetheless, these constructs exhibited increased engagement with all FcγRs which can be attributed to their higher avidity compared to their single Fc counterparts, and were also found to ameliorate symptoms of arthritis such as oedema and/or erythema of the paw in murine models [386]. Furthermore, they were also found to be effective in other models of autoimmune diseases, such as IPT [386], autoimmune myasthenia gravis [387] and inflammatory neuropathy [388]. Due to the success in *in vivo* models, Stradomers have also been produced on a human IgG1 background (GL-2045; Fig. 5c), and found to be effective in preventing CDC by sequestering C1q and inhibiting C5 convertase [389] and were more potent inhibitors of phagocytosis than IVIg [390]. Of note, GL-2045 (now known as PF 06755347) is currently undergoing Phase I clinical trials (NCT03275740).

The introduction of Fc mutations [391] or fusion of the Fc domain with the IgM tailpiece [392,393] may also be exploited to enhance the formation of hexameric structures upon antibody deposition (Fig. 5c). Fc hexamers developed on the human IgG1 background have been shown to be potent inhibitors of phagocytosis and also triggered internalisation and degradation of FcγRs [394]. However, they were also reported to show increased complement deposition. Complement deposition could be prevented by switching the isotype to human IgG4 background, while still retaining their ability to block phagocytosis [395]. Fc hexamers with anti-tumoral function have also been investigated, and shown potent anti-tumour responses *in vivo* [391,392].

Multimers featuring Fc domains arranged in tandem (tandem Fcs) have also been developed. One such example is Stradabodies, which have been investigated as a potential anti-cancer therapeutic. They we generated as an anti-epidermal growth factor receptor (EGFR) antibody on a human IgG1 background, which featured two Fc domains separated by an isoleucine zipper. These constructs were analysed *in vitro* using a HT29 cell line, with NK cells isolated from cancer patients. They demonstrated that the ADCC activity of the Stradabodies was significantly higher compared to the anti-EGFR unmodified antibody [32].

Other groups have also outlined the production of anti-CD20 constructs featuring three Fc domains. These constructs were found to be potent inducers of ADCC [29] and ADCP [30] against the Burkitt’s lymphoma cell line, Ramos, compared to their single Fc or double Fc counterparts. Moreover, levels of ADCP remained high in the presence of competing IgG [30], suggesting the increased avidity associated with the tandem architecture was efficacious at overcoming competition effects. Tandem Fcs specific to bacterial antigens have also been described, and have been associated with increased survival rate in Klebsiella lethal pneumonia models [31]. Notably, tandem Fc constructs have been reported to exhibit poor half-life *in vivo* [31], which may impede their progression into the clinic.

### 7.5. Inactivation of serum IgG using streptococcal antibody-degrading enzymes

Enzymatic inactivation of serum IgG is an additional potential strategy for improving monoclonal antibody therapies (Fig. 5d). As discussed in section 4, the enzyme IdeS from *S. pyogenes*, which inactivates IgG by cleaving within the lower hinge region, has been shown to enhance mAb efficacy, in both *in vitro* and *in vivo* murine models [2], due to its ability to rapidly cleave competing serum IgG. It is envisaged that the enzyme and therapeutic mAb are administered in a staggered way in order for the competing IgG and enzyme clear before administration of the IdeS-sensitive mAb.

*S. pyogenes* additionally secretes the enzymes Endoglycosidase S (EndoS) and Endoglycosidase S2 (EndoS2), which specifically remove the N-linked glycans from IgG Fc. Several variants of these enzymes have been developed for precise glycoengineering of IgG [396–398], which as previously discussed can alter immune effector function; however, the activity of these enzymes has also been demonstrated to aid in deactivation of pathogenic antibodies in various autoimmune disease models [399–401]. Moreover, IdeS and EndoS have been used collectively in murine allogenic bone marrow transplantation, for their ability to deactivate donor-specific antibodies [402]. Recent crystal structures of IgG1 Fc in complex with IdeS and EndoS reveal that they possess exquisite specificity towards human IgG due to their extensive interactions with the Fc globular domains [403] (Fig. 5d), an essential property for any biologic to avoid harmful off-target effects. This detailed structural information will likely aid in the development of variants that may allow for sequential dosing regimens, and may additionally help in the development of therapeutic antibodies resistant to modification by these enzymes. These enzyme-resistant antibodies may find utility in the treatment of streptococcal infections, and could potentially allow for co-administration of the enzyme and an antibody-based therapeutic [163].

## 8. Concluding remarks

The interaction of antibodies with Fc receptors is an important functional feature. However, the presence of competing IgG influences the capacity of an antibody to recruit the immune system. Understanding how competing IgG influences the activation threshold of a particular antibody is important in unleashing the full potential of an antibody in the therapeutic setting. The impact of competition effects is influenced by a wide range of factors within the microenvironment of the antibody, including features of the target, such as density, subclass, glycosylation status, together with the complexities of the effector function cell. Effective antibody therapeutics overcome or bypass serum competition effects.

## Figures and Tables

**Table 1:** Examples of Antibody-Like Proteins (ALPs) that may overcome competition effects from endogenous IgG†.

Format	Name	Target	Disease	Development stage	Citation

Antibody fragments and single domain antibodies	huCD64 × MHC II	FcγRI and MHC II	B-cell lymphoma	Preclinical	[20]
	AFM13	FcγRIIIA and CD30 (BiTe)	Hodgkin’s lymphoma	Phase 2 (NCT04101331)	[21]
	GTB-3550	FcγRIIIA, IL-15 and CD33 (TriKE)	Haematological malignancies	Phase 2 (NCT03214666)	[22]
Glyco-optimised mAbs	Mogamulizumab	CCR4	T cell lymphomas	Approved	[23]
	Obinutuzumab	CD20	Chronic lymphocytic leukemia and follicular lymphoma	Approved	[24]
	TrasGEX	HER2	Solid tumours	Phase 1 (NCT01409343)	[25,26]
Fc-optimised mAbs	Margetuximab	HER2	Breast cancer	Approved	[27,28]
	T3-Ab	CD20	Non-hodgkin lymphoma	Preclinical	[29,30]
Fc multimers	KP3- IgG-3Fc	MrkA KP3	*Klebsiella pneumoniae*	Preclinical	[31]
	Stradobody^™^	EGFR	Colon cancer	Preclinical	[32]

† C-C Motif Chemokine Receptor 4 (CCR4), human epidermal growth factor receptor 2 (HER2), epidermal growth factor receptor (EGFR), bispecific T Cell Engager (BiTe), trispecific killer cell engager (TriKE).

## Data Availability

No data was used for the research described in the article.
